# The force-dependent mechanism of DnaK-mediated mechanical folding

**DOI:** 10.1126/sciadv.aaq0243

**Published:** 2018-02-09

**Authors:** Judit Perales-Calvo, David Giganti, Guillaume Stirnemann, Sergi Garcia-Manyes

**Affiliations:** 1 Department of Physics and Randall Division of Cell and Molecular Biophysics, King’s College London, WC2R 2LS London, UK.; 2 CNRS Laboratoire de Biochimie Théorique, Institut de Biologie Physico-Chimique, Univ. Paris Denis Diderot, Sorbonne Paris Cité, PSL Research University, 13 rue Pierre et Marie Curie, 75005 Paris, France.

## Abstract

It is well established that chaperones modulate the protein folding free-energy landscape. However, the molecular determinants underlying chaperone-mediated mechanical folding remain largely elusive, primarily because the force-extended unfolded conformation fundamentally differs from that characterized in biochemistry experiments. We use single-molecule force-clamp spectroscopy, combined with molecular dynamics simulations, to study the effect that the Hsp70 system has on the mechanical folding of three mechanically stiff model proteins. Our results demonstrate that, when working independently, DnaJ (Hsp40) and DnaK (Hsp70) work as holdases, blocking refolding by binding to distinct substrate conformations. Whereas DnaK binds to molten globule–like forms, DnaJ recognizes a cryptic sequence in the extended state in an unanticipated force-dependent manner. By contrast, the synergetic coupling of the Hsp70 system exhibits a marked foldase behavior. Our results offer unprecedented molecular and kinetic insights into the mechanisms by which mechanical force finely regulates chaperone binding, directly affecting protein elasticity.

## Teaser

Mechanical force regulates the extent of chaperone binding to the protein substrate during mechanical folding.

## INTRODUCTION

Protein folding pathways typically involve the progressive burial of hydrophobic residues as the protein evolves toward the natively folded structure. Long solvent exposure of these hydrophobic patches—as a result of a slow conformational dynamics or of a highly crowded environment or due to a largely stabilized unfolded conformation (*1*)—can result in undesirable interactions that irreversibly compromise the folding process. To mitigate this threat, cells have evolved a limited number of chaperones that shield solvent-exposed reactive sites, minimizing misfolded conformations and/or biasing the protein toward the natively folded conformation (*2*). The role of chaperones is fundamental during cellular stress, induced, for example, by high temperatures or high oxidative stress conditions, often triggering undesired protein unfolding (*3*). Mechanical force is an alternative means to induce unfolded conformations, which, due to their extended nature, completely reveal otherwise concealed hydrophobic moieties to the environment. Chaperones are particularly important in physiological contexts where the extended conformation is relevant, such as during de novo folding in the ribosome, during membrane translocation (*4*), or in cardiomyocytes (*5*), constantly exposed to demanding cycles of protein unfolding and refolding under mechanical stress.

In general, chaperones can be classified according to their mechanism of action into “holdases”—those that do not use adenosine triphosphate (ATP) and bind with high affinity to the unfolded substrate to avoid aggregation while delaying folding—and “foldases,” which use the energy from ATP hydrolysis to effectively refold non-native polypeptides, often through multiple-step cycles of substrate binding and release. Hsp70 is the most abundant chaperone family (*6*), conserved across all domains of life. It carries out diverse and crucial functions, spanning protein folding, disaggregation of aggregates, and translocation across membranes (*3*). The general mechanistic aspects of the bacterial Hsp70, named DnaK, have been delineated through high-resolution structural snapshots (*3*). DnaK is composed of a C-terminal substrate-binding domain (*7*), which interacts with the unfolded polypeptide through a peptide-binding cleft. This binding interaction is allosterically regulated by the N-terminal nucleotide-binding domain (*8*), which is connected to the C-terminal domain by a flexible linker. Crucial to the adenosine triphosphatase (ATPase) role of DnaK is its close interplay with Hsp40 co-chaperones (such as the *Escherichia coli* DnaJ) and nucleotide exchange factors (such as the bacterial GrpE) (*6*).

The established canonical model for the DnaK machinery, uniquely combining both holdase and foldase roles, starts with the initial binding of unfolded proteins by DnaJ, which subsequently delivers it to DnaK (*6*). Rapid binding occurs in the ATP state of DnaK, whereby a flexible helical lid covering the binding cavity is in its open conformation. Stable peptide holding is accelerated by DnaJ binding onto DnaK, a process that stimulates the hydrolysis of ATP to adenosine diphosphate (ADP) and triggers the conformational closing of the DnaK C-terminal latch (*9*). Completion of the reaction cycle is catalyzed by GrpE, which induces the release of ADP from DnaK (*2*). Subsequent rebinding of ATP occurs concomitant to the dissociation of the DnaK-peptide complex (*10*).

Unlike other molecular machines, chaperones are generally promiscuous and show little specificity for substrates. For example, DnaK is known to target about five-residue-long hydrophobic patches, typically including leucine and isoleucine residues (*11*). Similarly, DnaJ is thought to recognize stretches of approximately eight residues enriched in aromatic and large hydrophobic aliphatic amino acids (*12*), although recent studies postulate a more specific binding consensus sequence (*13*), underscoring a putative higher degree of substrate specificity. Despite these structural advancements, the transient chaperone-substrate interaction and the highly dynamic conformational evolution defining the chaperone-assisted protein folding reaction pose experimental challenges that are difficult to address using traditional bulk techniques (*14*). Recent single-molecule experiments have begun to circumvent these limitations. For example, in single-molecule mechanical experiments, the applied force induces intermediate substrate-chaperone conformations that cannot be stabilized by other experimental means (*15*). In this vein, two recent nanomechanical experiments conducted in the traditional force-extension mode suggested the intriguing role of DnaJ as a foldase (*16*) and the avidity of DnaK for partially folded states (*17*). Despite this progress, our fundamental understanding of the (sub)molecular-scale mechanisms underlying the synergetic action of the different chaperones forming the DnaK system is still largely incomplete (*14*), especially due to the subtle and transient interactions between the co-chaperone(s) and the folding substrate (*3*). Particularly challenging are the mechanism of substrate recognition, the direct measurement of the kinetics of chaperone binding and unbinding, and the coupling between the chaperone activity with the fast conformational dynamics of the folding substrate, which is especially puzzling in the case of mechanical folding, because proteins need to fold from highly stretched conformations.

Force quench experiments using force-clamp atomic force spectroscopy enabled the monitoring of the individual (un)folding trajectories of single, mechanically resistant proteins under force (*18*). In this approach, mechanical unfolding drives the protein to an unfolded conformation close to its contour length. Such a low-entropy extended state is radically distinct from the more compact unfolded state typically described in folding experiments using chemical or thermal denaturants (*19*). Hence, mechanical unfolding brings the polypeptide to regions of the free-energy landscape that cannot be visited with ensemble biochemistry techniques (*19*, *20*). After mechanical unfolding, withdrawing the pulling force triggers protein refolding (*21*). Monitoring the individual folding trajectories from highly extended states uncovers the rich conformational dynamics of the single protein as it folds, using the length and mechanical stability of each conformation as its structural fingerprint (*18*, *22*). An important advantage of working under force-clamp conditions is that each protein conformation can be individually singled out, enabling us to individually probe its reactivity and kinetics.

Here, we use a single-molecule force-clamp spectroscopy assay to probe the effect that each of the co-chaperones forming the DnaK system (both individually and in combination) has on the folding conformational dynamics of three different well-characterized proteins [namely, ubiquitin (*18*) and the titin’s I27 (*21*) and Z1 immunoglobulin (Ig) domains (*23*)] that exhibit high mechanical stability yet largely distinct folding kinetics. Collectively, these experiments enable investigation of how the mechanical folding free-energy landscape is modulated by each molecular player forming the DnaK system.

Our results uncover the unexpected selectivity in conformational recognition for substrates for each co-chaperone; DnaJ binds the unfolded and extended protein form with high substrate specificity for a well-defined sequence that is cryptic within the structure of the client ubiquitin protein. Crucially, the interaction between the unfolded substrate and DnaJ is largely modulated by a force-dependent binding constant. By contrast, DnaK only recognizes the intermediate, collapsed conformations with high affinity yet low selectivity and, surprisingly, does not bind the extended (or the natively folded) conformations. From a broader perspective, our findings highlight the versatile role of the DnaK system; when working individually, both chaperones block folding, reminiscent of a “holdase” behavior. By contrast, when working in combination (and in the presence of GrpE), the DnaK system shows “foldase” activity, both by accelerating the folding of fast mechanical folder proteins, such as ubiquitin or titin I27, and by successfully refolding proteins that would otherwise not fold, such as the Z1 titin protein—possibly by avoiding protein misfolding. Together, our results extend the canonical view of the DnaK system and demonstrate a novel mechanism by which force modulates chaperone activity through fine-tuning its binding with the mechanically stretched protein substrate. This mechanism may have generic in vivo implications for the folding of proteins exposed to mechanical force.

## RESULTS

### DnaJ prevents ubiquitin refolding

**Fig. 1:**
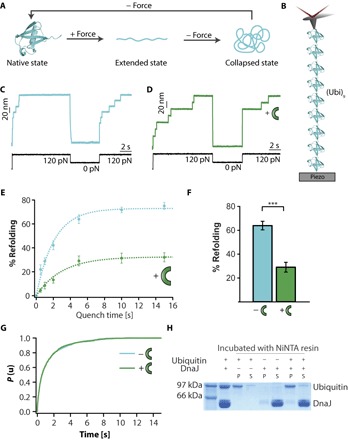
DnaJ binds to ubiquitin and blocks refolding. (**A**) Scheme of the distinct protein conformations visited by ubiquitin [Protein Data Bank (PDB): 1UBQ] when exposed to mechanical force, namely, native, collapsed, and extended (*22*). (**B**) Schematics of the single-molecule nanomechanical experiment, whereby a ubiquitin polyprotein (Ubi)_9_ is tethered between a gold substrate and an atomic force microscopy cantilever tip. (**C**) A force-quench protocol demonstrates that ubiquitin can quantitatively refold in *t*_q_ = 5 s, marked by the presence of 20-nm steps in the test pulse. (**D**) By contrast, upon addition of 5 μM DnaJ, the refolding efficiency is significantly decreased. (**E**) The kinetics of ubiquitin refolding is measured by changing *t*_q_, spanning the range 0.5 to 15 s (blue symbols; *t*_q_ = 0.5 s, *n* = 37 individual trajectories; *t*_q_ = 1 s, *n* = 56; *t*_q_ = 2 s, *n* = 36; *t*_q_ = 5s, *n* = 46; *t*_q_ = 10 s, *n* = 44; *t*_q_ = 15 s, *n* = 26). A single exponential fit to the refolding kinetics of wild-type ubiquitin displays a folding rate *k*_f_ = 0.52 ± 0.05 s^−1^. In the presence of DnaJ (green symbols), the refolding rate is decreased down to *k*_f_ = 0.29 ± 0.07 s^−1^ (*t*_q_ = 0.5 s, *n* = 36 trajectories; *t*_q_ = 1 s, *n* = 30; *t*_q_ = 2 s, *n* = 39; *t*_q_ = 5 s, *n* = 37; *t*_q_ = 10 s, *n* = 35; *t*_q_ = 15 s, *n* = 52). Such a reduced refolding rate can be explained in terms of the delayed ubiquitin collapse in the presence of DnaJ (fig. S1). (**F**) Whereas ubiquitin reaches a high refolding yield (~65%), the addition of DnaJ (green symbols) significantly (*P* < 0.0001) reduces the yield down to ~30% at a quench time of *t*_q_ = 5 s. (**G**) The unfolding kinetics of ubiquitin at 120 pN in the absence and presence of 5 μM DnaJ does not result in a change in the unfolding rate [*k*_u_ = 0.93 ± 0.06 s^−1^ (*n* = 195 unfolding trajectories) and *k*_u_ = 0.94 ± 0.07 s^−1^ (*n* = 138 unfolding trajectories), respectively], suggesting that DnaJ does not bind to the native state of ubiquitin. (**H**) These results are confirmed with pull-down assays, demonstrating that DnaJ and the folded ubiquitin do not coprecipitate (P, pellet; S, supernatant).

Dissecting the detailed molecular mechanisms by which each co-chaperone of the DnaK system individually modulates mechanical folding by recognizing specific conformations of the mechanically unfolded polypeptide ideally requires a model protein that exhibits well-characterized folding dynamics and that can spontaneously refold with high efficiency on relatively fast time scales. The small ubiquitin protein was well characterized in earlier force-clamp experiments, exhibiting defined folding states and kinetics (*18*). Specifically, during the mechanical folding trajectory from highly extended states, ubiquitin visits a structurally heterogeneous set of mechanically labile, molten globule–like collapsed conformations that are necessary intermediate precursors of the mechanically resistant native state (Fig. 1A) (*22*, *24*). In this context, we set out to investigate the separate effects that DnaJ and DnaK have on the dynamics of the folding of the model ubiquitin protein under force.

We started by probing, using a single-molecule force-clamp spectrometer, whether DnaJ has an effect on the mechanical folding of a ubiquitin polyprotein composed of nine identical repeats (Ubi)_9_ (Fig. 1B). Mechanical folding was probed using the force-quench approach, whereby an initial high-force pulse unfolds the protein, and after quenching the force for a limited period of time, the protein is restretched back again at high force (test pulse) to probe whether it properly refolded during the quench time. In the case of ubiquitin, applying a force of 120 pN triggered the 20-nm stepwise unfolding of the polyprotein (Fig. 1C). After full unfolding, the force was withdrawn for a long quench time (*t*_q_ = 5 s) to allow refolding. The success of the folding reaction was evaluated in the test pulse, whereby the protein was stretched back at 120 pN, resulting in a 20-nm stepwise elongation, which certified that the protein had regained mechanical stability—a direct proxy for successful refolding. For each trajectory, the refolding efficiency was calculated as the ratio between the number of 20-nm steps present in the test and initial pulses. The high refolding efficiency of ubiquitin contrasted with the much lower occurrence of 20-nm steps in the test pulse upon addition of 5 μM DnaJ (Fig. 1D). Direct comparison of the kinetics of ubiquitin refolding (Fig. 1E, blue symbols) as a function of the quench time in the range *t*_q_ = 0.5 to 15 s with that obtained in the presence of 5 μM DnaJ (Fig. 1E, green symbols) revealed that the refolding yield of ubiquitin plateaus at a high (~75%) refolding yield value, exhibiting a folding rate constant of *k*_f_ = 0.52 s^−1^, in accordance with previous results (*22*). By contrast, in the presence of DnaJ, the plateau in the refolding kinetics is capped at ~30%. Such a stark reduction in the refolding efficiency (Fig. 1F) underpins an effective binding interaction between DnaJ and ubiquitin.

The next step was to investigate which of the distinct ubiquitin conformation(s)—native, extended, or collapsed (Fig. 1A)—is recognized by DnaJ. Although DnaJ’s main recognized role is to bind non-native polypeptides, it has also been shown to bind the native state of several proteins, including σ^32^, DnaB (*25*), and RepE (*26*). To directly test whether DnaJ recognizes the folded state of ubiquitin, we measured its mechanical unfolding rate when pulled at 120 pN, in the absence (Fig. 1G, blue line, *k*_u_ = 0.93s^−1^) and in the presence of DnaJ (Fig. 1G, green line, *k*_u_ = 0.94 s^−1^). The close agreement in the measured unfolding rates strongly suggests that DnaJ does not bind native ubiquitin. Although ligand binding typically results in a change of the mechanical properties of the protein substrate (*27*)—hence altering its unfolding rate—it is still possible that DnaJ binding is not directly reflected in a change of the mechanical properties of native ubiquitin. Parallel pull-down experiments confirmed that DnaJ does not coprecipitate with ubiquitin (Fig. 1H). Combined, these findings imply that, in our experiments, DnaJ binds a peptide sequence that is cryptic within the protein’s native structure and that gets exposed to the solution only after mechanical unfolding.

### DnaJ binds with high affinity to the mechanically extended ubiquitin

**Fig. 2:**
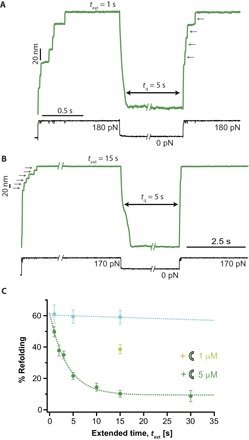
DnaJ binds to the unfolded and extended state of ubiquitin. (**A**) Changing the time of the initial pulse in the presence of DnaJ enables binding to the unfolded ubiquitin. After a short extended time *t*_ext_ = 1 s, ubiquitin shows a high refolding efficiency, fingerprinted by a large number of 20-nm steps in the test pulse. (**B**) Upon increasing *t*_ext_ = 15 s, the refolding efficiency is markedly decreased. (**C**) Evolution of the refolding efficiency as a function of *t*_ext_ for a constant *t*_q_ = 5 s. In the presence of 5 μM DnaJ, the folding efficiency decreases exponentially with the *t*_ext_ with an associated rate *k* = 0.27 ± 0.05 s^−1^ (green symbols). The number of individual trajectories used was *t*_ext_ = 1 s, *n* = 33; *t*_ext_ = 2 s, *n* = 43; *t*_ext_ = 3 s, *n* = 57; *t*_ext_ = 5 s, *n* = 84; *t*_ext_ = 10 s, *n* = 52; *t*_ext_ = 15 s, *n* = 45; *t*_ext_ = 30 s, *n* = 21. Decreasing the concentration of DnaJ down to 1 μM increases the refolding percentage for a constant *t*_ext_ = 15 s (*n* = 63, light green symbol), demonstrating that DnaJ binding to ubiquitin is a concentration-dependent mechanism with an associated association constant, *k*_on_. In the absence of DnaJ, the refolding of ubiquitin is largely independent of *t*_ext_ (blue symbols; *t*_ext_ = 1 s, *n* = 26; *t*_ext_ = 5 s, *n* = 33; *t*_ext_ = 15 s, *n* = 17).

To probe whether DnaJ binds the unfolded state instead, we modified the force-quench protocol by measuring the refolding yield after systematically changing the time, *t*_ext_, the protein is left unfolded and extended at high force in the presence of 5 μM DnaJ. Although leaving the protein extended for a short *t*_ext_ = 1 s resulted in a high refolding efficiency, hallmarked by a large number of 20-nm steps present in the test pulse (arrows in Fig. 2A), increasing *t*_ext_ = 15 s markedly decreased refolding (Fig. 2B). Systematic analysis of the refolding yield by changing *t*_ext_ within the range spanning *t*_ext_ = 1 to 30 s for a constant *t*_q_ = 5 s revealed that the refolding efficiency decreased exponentially with increasing *t*_ext_, with an associated characteristic decay time of *k* = 0.27 s^−1^ (Fig. 2C, green symbols). The same behavior, albeit to a lower extent, is observed when using a lower DnaJ concentration (1 μM) for a *t*_ext_ = 15 s (Fig. 2C, light green symbol). Protein collapse is significantly slowed down as *t*_ext_ is increased (fig. S1), further suggesting that chaperone binding affects protein dynamics. Control experiments in the absence of DnaJ showed that ubiquitin refolding is almost independent on *t*_ext_ (and only dependent on *t*_q_; Fig. 2C, blue circles). Together, these results suggest that longer exposure of the unfolded ubiquitin to the solvent promotes DnaJ binding with a binding rate, *k*_on_. Crucially, DnaJ binding significantly reduces the refolding yield. Even at long exposure times, folding was not completely abolished (a residual 10% refolding was observed), suggesting that the DnaJ-ubiquitin interaction is in equilibrium between the chaperone-bound and unbound populations.

### The substrate primary sequence dictates DnaJ binding

Earlier experiments that screened cellulose-bound peptides for DnaJ binding revealed that the affinity of DnaJ for non-native peptides is rather unspecific, mostly recognizing the side chains of a continuous stretch of eight amino acids, enriched in aromatic and also in large hydrophobic aliphatic residues and arginine (*12*). Follow-up computationally driven studies on the Hsp40 yeast Ydj1 protein identified a unique substrate-binding motif characterized by a well-defined consensus sequence, GX[LMQ]{P}X{P}{CIMPVW}, where [XY] denotes either X or Y and {XY} designates neither X nor Y (*13*). These experiments provided the structural basis of substrate recognition, occurring in the hydrophobic pocket located on the peptide-binding fragment of type I Hsp40s. The ubiquitin substrate used in our experiments displays such a consensus sequence between positions 47 and 53 (GKQLEDG). Hence, it is tempting to speculate that DnaJ binding to the mechanically unfolded state of ubiquitin is highly specific. Alternatively, it is also possible that the binding of DnaJ to hydrophobic regions exposed upon mechanical unfolding is less sequence-specific, underlying a more common mechanism that underpins the avidity of the chaperone for unfolded substrates. The most obvious strategy to test the generality of DnaJ binding to mechanically unfolded states and its sequence specificity would be to introduce point mutations in the consensus sequence of ubiquitin. However, this proved challenging because all tested ubiquitin mutants exhibited impaired folding (fig. S2). Instead, we repeated the experiments with a different protein lacking the consensus sequence but well characterized in mechanical folding assays, the I27th Ig domain of titin (*21*). Our single-molecule results revealed that, similar to ubiquitin, the refolding efficiency of I27 is highly compromised in the presence of 5 μM DnaJ (fig.S3, A to C). However, although DnaJ did not bind the native state of I27 (fig. S3D), it did not bind the I27 unfolded and extended state (fig. S3E), in stark contrast to ubiquitin. These results strongly suggest that DnaJ specifically recognizes the mechanically unfolded state of ubiquitin through the proposed consensus sequence.

### DnaJ binding to unfolded ubiquitin is force-dependent

**Fig. 3:**
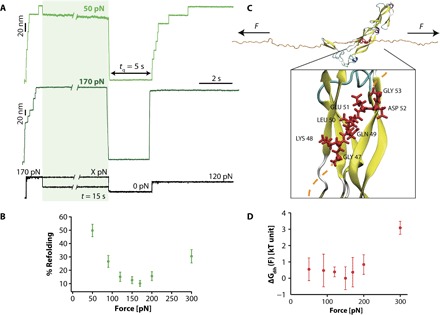
DnaJ binding to the unfolded ubiquitin is force-dependent. (**A**) Changing the force at which ubiquitin is left stretched after mechanical unfolding results in a marked change in the refolding yield. In these experiments, a high force of 170 pN for a short time *t* = 1 s triggers ubiquitin unfolding. The force is then varied within the range *X* = 50 to 300 pN for a period *t* = 15 s before a *t*_q_ = 5 s pulse is applied to trigger protein refolding. The refolding efficiency is tested in the test pulse, when the protein is stretched back at 120 pN. (**B**) Dependency of the refolding percentage with the pulling force (*F* = 30 pN, *n* = 33; *F* = 50 pN, *n* = 30; *F* = 90 pN, *n* = 25; *F* = 120 pN, *n* = 36; *F* = 150 pN, *n* = 28; *F* = 170 pN, *n* = 45; *F* = 200 pN, *n* = 43; *F* = 300 pN, *n* = 22). (**C**) Snapshot of the interaction of DnaJ (PDB: 1NLT) with the unfolded and mechanically stretched ubiquitin (orange ribbon): The interaction surface involves seven residues (red), exhibiting the consensus sequence GKQLEDG. Inset: Zoom of the reconstructed (see Materials and Methods) DnaJ-ubiquitin fragment system. The bonds of the amino acids from the consensus sequence are shown as red tubes, and the corresponding C_α_’s are shown as red balls. (**D**) Five couples (ϕ, ψ) of backbone dihedral angles can be defined for the consensus sequence to estimate the free-energy contribution corresponding to the overall energetic cost underlying the force-induced remodeling of the dihedral angles of the ubiquitin interaction fragment upon DnaJ binding (see Materials and Methods and the Supplementary Materials for details about the calculation). Error bars correspond to the SDs observed among the five independent runs at each force, and the data are normalized with respect to the lowest free-energy value (observed at 150 pN).

Given that the application of force crucially reduces protein flexibility required for binding, we next posed the question of whether mechanical force, which adjusts the dynamics of the protein backbone, regulates chaperone binding. To this goal, we modified the force-quench protocol, whereby after the first short (1 s) pulse at 170 pN that triggered ubiquitin unfolding, the second pulse at varying forces (spanning 50 to 300 pN) was applied for 15 s, enabling the binding of DnaJ to a series of energetically similar stretched conformations that only differ in the geometry and dynamics of the backbone dihedral angles (Fig. 3A). The refolding success was assessed in each case in the test pulse, in which the protein was restretched at a high force (120 pN) after a constant *t*_q_ = 5 s refolding time. Figure 3B shows that, surprisingly, the refolding efficiency is largely modified by the stretching force in a nonmonotonous manner, whereby the refolding percentage is higher at low (50 pN, ~50%) and high forces (300 pN, ~30%). The minimum refolding (10%) occurs at intermediate forces (150 to 170 pN), where binding is optimum.

To rationalize this unexpectedly complex behavior, we used molecular dynamics (MD) simulations in explicit solvent to explore the molecular mechanisms accounting for the drastic variations in DnaJ binding to structurally distinct force-induced conformations of the mechanically stretched ubiquitin (Fig. 3C). We applied a previously devised methodology (*28*) designed to study the structural and dynamical molecular details that described the force-dependent changes in ubiquitin flexibility in terms of the response of each backbone dihedral angle to the stretching force dictated by the worm-like chain model of polymer elasticity. Here, we extended this approach to estimate, at a given force, the energetic cost to bring each dihedral angle from its stable value in the “free”-ubiquitin fragment under a given force to its new constrained conformation imposed by DnaJ binding (Supplementary Methods and fig. S4). Noticeably, the calculated free-energy values associated with the overall force-induced remodeling of the dihedral angles corresponding to the binding between the DnaJ-ubiquitin fragment (inset in Fig. 3C) also display a maximum energy change (3 *k*_B_T) at high forces ~300 pN (Fig. 3D). To obtain a structural interpretation for the obtained values, we constructed, for each interacting residue, the Ramachandran plot at each force and compared the position of each side chain in the free and bound conditions (fig. S5). The seven-residue consensus sequence allows us to define five couples of (φ, ψ) dihedral angles that are constrained by the binding to DnaJ, namely, from LYS48 to ASP52. We observed that LYS48, GLU51, and ASP52 display dihedral angle values that are favored at low forces, because they correspond to Ramachandran regions that are populated in the absence of force. By contrast, GLN49 and LEU50, participating in the β-sheet motif, exhibit dihedral angle values that are favored at high forces (top left corner). Qualitatively, the graph shown in Fig. 3D, globally recapitulating the experimental results, can be rationalized by the observation that, at low forces, there is a very large free-energy cost to stretch the dihedral angles of GLN49 and LEU50 toward their corresponding values. Conversely, at high forces, there is a cost to compress the dihedrals of LYS48, GLU51, and ASP52 toward lower values. Hence, it is only at intermediate forces that these residues are in intermediate positions, overall resulting in the best energetic compromise.

### DnaK binds to ubiquitin refolding intermediates

**Fig. 4:**
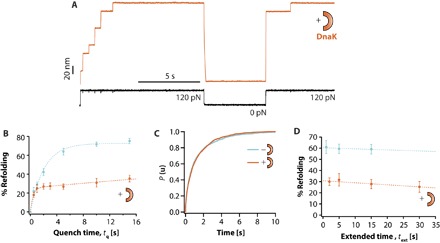
DnaK(ADP) hinders protein folding by binding to the intermediate collapsed states. (**A**) Refolding of ubiquitin is severely compromised in the presence of 5 μM DnaK(ADP). (**B**) The associated refolding kinetics (orange symbols; *t*_q_ = 0.5 s, *n* = 30 individual trajectories; *t*_q_ = 1 s, *n* = 46; *t*_q_ = 2 s, *n* = 43; *t*_q_ = 5 s, *n* = 51; *t*_q_ = 10 s, *n* = 27; *t*_q_ = 15 s, *n* = 31) plateaus at a low refolding yield of ~30% and cannot be captured by a single exponential. Instead, a calculated function obtained from a multiparametric fit (see Materials and Methods and figs. S11 and S12) reproduces the experimental data with high accuracy (orange fit). (**C**) The kinetics of ubiquitin unfolding at 120 pN (*k*_u_ = 0.93 ± 0.06 s^−1^, *n* = 195 unfolding trajectories) is not affected upon addition of DnaK (*k*_u_ = 0.92 ± 0.05 s^−1^, *n* = 194 unfolding trajectories), hence suggesting that DnaK does not bind the native state of ubiquitin. (**D**) Similarly, the refolding yield is not significantly modified with *t*_ext_ (*t*_ext_ = 2 s, *n* = 49; *t*_ext_ = 5 s, *n* = 21; *t*_ext_ = 15 s, *n* = 22; *t*_ext_ = 30 s, *n* = 26). Combined, these observations suggest that DnaK binds the intermediate, collapsed conformations.

We next moved on to dissecting the role of DnaK on the mechanical folding of ubiquitin. When ubiquitin was exposed to 5 μM DnaK in the presence of ADP—hence in its closed state, showing high affinity for substrates—the individual folding trajectories showed a drastic reduction in the refolding efficiency (Fig. 4A). Similar to DnaJ, the refolding kinetics obtained for DnaK(ADP) at varying *t*_q_ = 0.5 to 15 s plateaus at a low (~30%) refolding yield (Fig. 4B). However, in contrast to DnaJ, the shape of the refolding kinetics as a function of quench time, *t*_q_, cannot be captured by a single exponential (fig. S6). The refolding kinetics is fast at low *t*_q_ values (up to *t*_q_ ~ 1 s), after which the refolding evolution with time slows down, resulting in a much shallower dependence with *t*_q_, reminiscent of a more complex kinetics. Together, these results demonstrate that DnaK(ADP) alone also prevents quantitative ubiquitin refolding.

Following the same approach used for DnaJ, we investigated the substrate conformation(s) that is recognized by DnaK. Similar to DnaJ, DnaK does not bind the native state of ubiquitin (Fig. 4C). This is further confirmed by the observation that DnaK does not increase its ATPase activity upon incubation with ubiquitin (fig. S7). However, and in stark contrast to DnaJ, DnaK does not bind the unfolded state either (at least at the tested pulling force of 120 pN), because the refolding yield is practically unaffected when increasing *t*_ext_ (Fig. 4D). Hence, our experiments strongly suggest that DnaK exclusively recognizes the intermediate collapsed states of ubiquitin. Similarly, DnaK recognizes the collapsed states of I27 as well (fig. S8), resulting in the blocking of I27 refolding. Although DnaK(ADP) drastically hampers ubiquitin refolding (~30%), the effect is less pronounced in I27, where the refolding yield is higher (~50%). These results suggest that either the affinity of DnaK is higher for ubiquitin (thus exhibiting a higher *k*_on_) or, alternatively, DnaK can dissociate from I27 faster (higher *k*_off_), allowing the protein to successfully refold.

The versatility of the force protocols used in our experimental approach offer the so-far elusive possibility of quantifying the binding constants of each independent co-chaperone to each distinct conformation of the protein substrate. Quantification is based on the experimental evidence that substrate domains lose their ability to refold upon binding DnaJ or DnaK(ADP) with an associated binding constant, *k*_on_. This situation can be reversed in the case the chaperone dissociates (*k*_off_). On the basis of the experimental results, we implemented a kinetic model based on ordinary differential equations to evaluate the on and off rates of each chaperone to each polyprotein placed under force and compute how the different protein populations (folded, unfolded-free, unfolded-bound, collapsed-free, and collapsed-bound) evolve over time as the applied force is changed in our experiments (figs. S9 and S10 and table S1). The multiparametric fitting of the data (i) reproduces the experimental folding kinetics of ubiquitin in the presence of DnaJ and DnaK, (ii) enables quantification of the dissociation constant (*K*_d_ = *k*_off_/*k*_on_) for each chaperone/protein conformation tandem (fig. S9), and (iii) predicts the force dependency of binding observed experimentally (fig. S10). In particular, the best fit to the data confirms that, in the case of ubiquitin, DnaJ binds to the extended state with high affinity (*K*_d_ = 9.4 × 10^−7^ M, table S1). By contrast, DnaK underscores a high affinity for the collapsed states (*K*_d_ = 1.4 × 10^−7^ M) and an almost absence of binding to the extended state. The situation is significantly reversed in the case of I27, whereby there is no binding of DnaJ to the stretched conformation but a high affinity for the collapsed states instead (*K*_d_ = 3.3 × 10^−7^ M), especially due to a high *k*_on_ = 2.5 × 10^5^ M^−1^ s^−1^. Similarly, DnaK shows a comparable affinity to the collapsed states (*K*_d_ = 2.7 × 10^−7^ M).

### The complete DnaKJE system increases refolding efficiency in ubiquitin

**Fig. 5:**
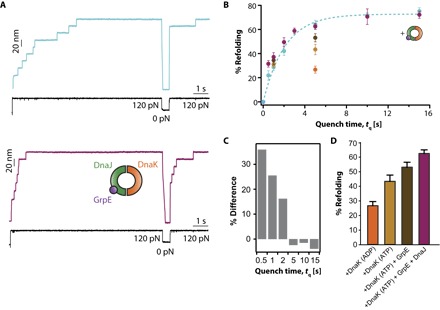
The complete DnaKJE system enhances ubiquitin refolding. (**A**) Individual folding trajectories corresponding to low *t*_q_ = 0.5 s reveal that the addition of the complete DnaKJE system improves ubiquitin refolding. (**B**) Comparison of the refolding kinetics of ubiquitin in the absence (blue symbols) and the presence (violet symbols; *t*_q_ = 0.5 s, *n* = 69 individual trajectories; *t*_q_ = 1 s, *n* = 32; *t*_q_ = 2 s, *n* = 40; *t*_q_ = 5 s, *n* = 46; *t*_q_ = 10 s, *n* = 17; *t*_q_ = 15 s, *n* = 28) of the DnaKJE system. Single exponential fit to the data demonstrates that the rate of refolding *k*_f_ = 0.52 ± 0.05 s^−1^ is also increased in the presence of the DnaK system (*k*_f_ = 0.92 ± 0.1 s^−1^). (**C**) As a consequence of this faster folding rate, at short quench times *t*_q_ = 0.5 to 2 s, the folding increase is more pronounced [*P* = 0.064 (*t*_q_ = 0.5 s); *P* = 0.01163 (*t*_q_ = 1 s); *P* = 0.0346 (*t*_q_ = 2 s)] when compared to longer *t*_q_ values. (**D**) Comparison of the refolding percentage for a given *t*_q_ = 5 s highlights the additive role of each of the molecular components of the DnaKJE system [DnaK(ADP) in orange, *n* = 51 trajectories; DnaK(ATP) in light brown, *n* = 40; DnaK(ATP) + GrpE in dark brown, *n* = 36; and DnaK(ATP) + GrpE + DnaJ in violet, *n* = 46].

So far, our experiments demonstrate that, when working individually, both DnaJ and DnaK chaperones recognize different conformations of ubiquitin (whereas they compete for the same collapsed intermediate conformations of I27) and significantly diminish the refolding yield. It remains to be seen what the effect of the two co-chaperones working in synergy would be on model fast folders such as ubiquitin, which successfully refold on their own. Figure 5 demonstrates that, in the presence of the DnaKJE system, the refolding efficiency is completely restored (Fig. 5, A and B). At short quench times (*t*_q_ ~ 0.5 s), the refolding efficiency is increased (*P* < 0.005) compared to the refolding percentage of ubiquitin obtained in the absence of chaperone (Fig. 5C). Moreover, the refolding rate is significantly increased (*k*_f_ = 0.92 s^−1^ versus *k*_f_ = 0.52 s^−1^). To rule out that this effect might be due to crowding or nonspecific effects, we repeated the experiments with 15 μM bovine serum albumin (BSA) (fig. S11), resulting in a folding kinetics that overlaps that of ubiquitin in the absence of chaperones, thus demonstrating the specificity of the DnaKJE chaperone system. Finally, to dissect the contribution of each molecular player of the DnaKJE system to the ubiquitin refolding kinetics, we compared the folding efficiency for a given *t*_q_ = 5 s when each element of the system (+ATP, + GrpE, +DnaJ) was sequentially added to the measuring DnaK solution (Fig. 5D and fig. S12). The addition of each component of the DnaK system improves the folding yield, confirming that each of the components has an essential role in the mechanical refolding process and is required to reach the maximum refolding efficiency.

Overall, the results shown in Fig. 5 demonstrate that the DnaKJE system can increase the folding kinetics of model fast folders that can readily refold on their own, such as ubiquitin. The enhancement of the refolding percentage (and its kinetics) by the DnaKJE system is even more evident for the I27 model protein, especially at low *t*_q_ values, where the folding efficiency can increase by as much as 40% (*P* < 0.0001) at *t*_q_ = 0.5 s (fig. S13).

### The complete DnaKJE system works as a general folding enhancer

To probe the generality of the catalytic effect of the DnaKJE system on mechanical folding, we investigated how the addition of the whole DnaKJE machinery modifies the folding efficiency of a polyprotein made of identical repeats of the titin Z1 Ig domain, (Z1)_8_, which harbors a physiological mechanical role. In this case, application of a force of 100 pN triggered the 25-nm stepwise unfolding of the (Z1)_8_ polyprotein (fig. S14A) (*23*). After a force quench of *t*_q_ = 5 s, the protein was stretched back at 100 pN. In most of the cases, the elongation of the protein back up to the fully unfolded state lacked the 25-nm steps, implying that the protein remained unfolded and devoid of mechanical stability, akin to an entropic spring (fig. S14B). The addition of the entire DnaKJE system (5 μM DnaK + 5 μM DnaJ + 1 μM GrpE + 5 mM ATP, fig. S14C) markedly increased the yield of Z1 refolding from ~15 to ~35% (fig. S14, D and E), demonstrating that the ATP-dependent DnaK machinery can enhance the refolding of the otherwise folding-incompetent Z1 Ig domain.

## DISCUSSION

Advanced structural techniques have provided a very good generic mechanistic understanding of the main molecular motions underlying the action of the DnaKJE system, describing the precise interactions within the interacting co-chaperones and between each co-chaperone and the client substrates. For example, it was recently beautifully demonstrated (*29*) that the lid of DnaK can visit different conformational states; whereas the closed conformation might encase extended peptide stretches of unfolded proteins and nascent chains, a more flexible and open conformation can accommodate regions with substantial tertiary structure, including natively folded and aggregated proteins. Follow-up single-molecule experiments further demonstrated that DnaK recognizes partially folded and near-native conformations (*17*).

**Fig. 6:**
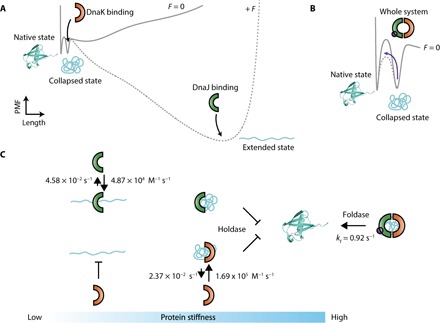
Schematic representation of the effect of the DnaK system on the one-dimensional projection of the folding free-energy landscape under force and its associated folding kinetics. (**A**) One-dimensional representation of the force modulation of the ubiquitin free-energy landscape, highlighting the different conformations (native, collapsed, and extended) (*31*). While DnaJ binds the unfolded and extended state, preventing protein refolding, DnaK stabilizes the collapsed conformations instead. (**B**) By contrast, the whole DnaKJE complex catalyzes the collapsed–to–native state transition. (**C**) Kinetic scheme highlighting the dynamic interaction—hallmarked by the related binding (*k*_on_) and dissociation constants (*k*_off_)—between each chaperone and the distinct conformation of the ubiquitin substrate (at a force of 170 pN for the extended conformation). The protein-chaperone interaction has direct implications for protein elasticity.

Our experiments broadly demonstrate the conformational diversity of the client substrates recognized by each of the different chaperone partners conforming the DnaKJE system. Whereas DnaJ recognizes the unfolded and extended state of ubiquitin (probably through a specific consensus sequence), DnaK recognizes the collapsed, partially folded conformations instead (conceivably through the hydrophobic patches that match the reported recognition sequences, fig. S15) (*11*). When working independently, the binding of each chaperone to protein substrates (such as the mechanically stable models ubiquitin and I27 proteins) drastically prevents protein folding, hindering the evolution of the ensemble of collapsed conformations into the native state. In this sense, both chaperones work as a holdase, implying that they keep the substrate protein unfolded, possibly not only avoiding further aggregation but also preventing successful mechanical refolding, hence resulting in a mechanically compliant polypeptide. This behavior is reminiscent of that of SecB, which binds to the molten globule conformation and prevents the formation of stable tertiary contacts when maltose binding protein is used as a substrate (*30*). By contrast, when both DnaJ and DnaK co-chaperones work in synergy, together with GrpE and in the presence of ATP, they can efficiently revert their holdase role and turn it into an effective foldase. The DnaKJ system increases the refolding rate of proteins that typically refold on their own (such as ubiquitin and titin’s I27 Ig domain) (*22*) and crucially promotes folding of otherwise folding-inefficient substrates such as titin’s Z1 Ig domain, together resulting in stiff and mechanically stable protein forms. Hence, the DnaKJE system exhibits a versatile foldase mechanism, adaptable to different protein substrates. The distinct roles of each of the co-chaperones in modulating protein folding underpin a complex, fine-tuned alteration of the folding energy landscape (*31*) of each mechanically unfolded polypeptide according to its (force-driven) conformation and primary sequence (Fig. 6).

An important finding in our experiments is the discovery of the important role that the unfolded and extended proteins form in guiding and modulating chaperone-mediated mechanical protein folding. The in vivo relevance of these extended conformations seems to be increasingly important, because mechanical unfolding is at the core of several biological processes such as the proteasome-mediated degradation of proteins by the bacterial ClpX (*32*). The mitochondrial import motor (*33*) relies on the matrix Hsp70 chaperone, which functions by both pulling and holding the imported proteins. Similarly, the Hsp70 system works in cooperation with Hsp100 disaggregases such as the bacterial ClpB, applying mechanical forces that can pull individual polypeptides from an aggregated mesh, thus reversing and resolving misfolded conformations (*3*). Perhaps more paradigmatic examples entail proteins that bear per se an important mechanical function, such as those involved in tissue elasticity (for example, the giant titin protein). In this vein, down-regulation of heat shock proteins, including Hsp27, Hsp70, and others, is tightly related to cardiac diseases (*5*, *34*). Hence, it is interesting to speculate a more ubiquitous scenario whereby the Hsp70 system recognizes mechanically unfolded conformations, most likely avoiding their misfolding and hence directly or indirectly modulating their refolding rate. Particularly enticing in this context is our observation that local backbone conformations (tuned with mechanical force) emerge as a key modulator of DnaJ binding to extended proteins, with a massive effect on its folding efficiency. The tight interplay between the applied force and the chaperone-protein interface establishes a novel structure-force paradigm, whereby mechanical tension fine-tunes the local protein conformation amenable to chaperone binding. Hence, it is tempting to propose a novel folding scenario whereby small variations in the force applied to the substrate (thus finely modulating its conformation) can have large implications on the folding fate of the protein.

On the other hand, the elongated state of the protein is also encountered in the vectorial folding in the ribosome, thus playing an important role in the energy landscape governing de novo folding (*35*). Given the physical constraints of the exit channel of the large ribosomal subunit, which is 100 Å long and has a diameter of <10 Å at its narrowest end, the nascent polypeptide is thought to be almost unfolded, and productive folding occurs only once it has fully emerged from the ribosome (*36*). Hence, given the slow translation rate, proteins visit partially unfolded conformations—thus risking misfolding and aggregation—for large periods of time. For this reason, ribosome-bound chaperones stabilize the nascent chains and protect them from non-native interactions. For example, the bacterial trigger factor (TF) binds to the titin I27 protein upon translation until the entire sequence has exited the ribosome and is competent for folding (*37*). The extended protein emerging from the ribosome is exposed to mechanical forces (*38*). Elegant single-molecule experiments demonstrated how pulling forces of >12 pN generated by the collapse and folding of the already translated protein can resume translation of the ribosome-stalled SecM nascent chain that still lies in the exit tunnel (*39*). Our results suggest that the substrate primary sequence could play a fundamental role in the recognition of largely unfolded substrates by DnaJ. In this vein, the consensus sequence could be the signal for DnaJ to quickly attach polypeptides that are being synthesized in the ribosome and kept unfolded by the TF. After protein recruitment, both DnaJ and DnaK can bind to partially (un)folded intermediates, thereby activating the foldase machinery, which might culminate in successful substrate refolding.

On a broader context, our experiments add new conceptual advancement on the regulation of protein activity upon force-regulated exposure of cryptic sites. Solvent exposure of peptide sequences buried in the native fold by mechanical unfolding has emerged as an important mechanism in the activation of several proteins involved in focal adhesions, such as talin, which can only bind vinculin when mechanically stretched (*40*). Similarly, binding of vinculin to α-catenin also requires mechanical unfolding, making the binding site accessible for binding (*41*). From the mechanochemistry perspective, force-exposure of structurally buried disulfide bonds or reactive side chains triggers a variety of posttranslational modifications that regulate protein folding and elasticity (*42*, *43*). Here, we add chaperone binding to mechanically exposed cryptic sequences as a key regulator of mechanical folding.

## MATERIALS AND METHODS

### Protein purification

All polyproteins used as substrates—(I27)_8_, (ubiquitin)_9_, and (Z1)_8_—were cloned into the pQE80L (Qiagen) expression vector and transformed into the BLR (DE3) *E. coli* expression strain. Cells were grown in LB supplemented with ampicillin (100 μg/ml) at 37°C. After reaching an OD_600_ (optical density at 600 nm) of ~0.6, cultures were induced with 1 mM isopropyl-β-d-thiogalactopyranoside and incubated overnight at 37°C (I27 and ubiquitin) or 20°C (Z1). Cells were disrupted with a French press, and the polyproteins from the lysate were purified by metal affinity chromatography on Talon resin (Takara, Clontech) followed by gel filtration using a Superdex 200 10/300 GL column (GE Biosciences). Proteins were stored in phosphate-buffered saline (PBS) buffer at 4°C. DnaJ, DnaK, and GrpE were expressed in BL21(DE3) cells and purified as described elsewhere (*44*).

### Single-molecule force-clamp spectroscopy experiments

Single-molecule experiments were conducted at room temperature using both a homemade setup described elsewhere and a commercial Luigs and Neumann force spectrometer (*45*). Each protein sample was prepared by depositing 1 to 5 μl of protein (at a concentration of 0.5 to 1.5 mg/ml) onto a freshly evaporated gold cover slide. All the experiments were carried out using PBS + 5 mM MgCl_2_ buffer at pH 7.4. The concentrations of chaperones used were 5 μM DnaJ_2_, 5 μM DnaK, and 1 μM GrpE. For the experiments with DnaK(ATP), the buffer was supplemented with a regenerator system composed of 5 mM ATP, 5 mM phosphoenolpyruvate, and pyruvate kinase (15 μg/ml). Each cantilever (Si_3_N_4_ Bruker MLCT-AUHW) was individually calibrated using the equipartition theorem, yielding a typical spring constant of ~15 pN/nm. All the experiments were performed at a constant temperature of 22° ± 2°C. Single proteins were picked out from the surface with a constant force of ~1 nN during 1 to 2 s to promote the nonspecific adhesions of the proteins to the cantilever surface. Then, the piezoelectric actuator was retracted by applying a pulling force. If a tether had been formed, the force rapidly (<3 ms) stabilized at the set point. The error bars were calculated by bootstrapping the values observed for all the trajectories considered for each particular condition.

### Data analysis

All data were recorded and analyzed using custom software written in Igor Pro 6.32 (WaveMetrics). To measure the unfolding kinetics, we selected traces with six or more unfolding events exhibiting a long detachment time. These traces were summed, averaged, and normalized. The resulting normalized probability of unfolding *P*(u) was fitted to a single exponential to obtain the unfolding rate for each particular force and protein substrate. To estimate the error of our experimentally obtained rate constants, we used the nonparametric bootstrap method. This process was repeated 500 times for each experimental condition. In our refolding studies, we used traces containing five or more unfolding steps in the initial pulse. Only traces showing equal extension at the end of the initial pulse and the end of the test pulse were included to ensure that the same protein was stretched in the two pulses. The refolding percentage for each condition was calculated as the ratio between the number of unfolding events in the test and initial pulses. In our refolding experiments, we (i) counted the number of independent observations *n* corresponding to the total number of protein domains that unfolded in the test pulse and (ii) compared with the number of successfully refolded domains observed in the denature pulse. The mean and the SEM for fractions were estimated through the bootstrap method (*46*), where each recording was treated as an independent data point. SEM for fit parameters was determined as the SE for the coefficient in the fit, given the measurement errors of the individual data points. The bootstrap method (measuring the observed error) provided similar yet higher error bars than the binomial distribution (providing information on the expected error).

### Pull-down experiments

Ubiquitin (His-tagged) (10 μM) and DnaJ (10 μM) were incubated in PBS + 5 mM MgCl_2_ during 1 hour at room temperature. The samples were mixed with NiNTA beads for 90 min at 4°C with gentle shaking in the same buffer. Unbound proteins were removed by centrifugation (supernatant). The resin was washed three times, and pellets were eluted with 300 mM imidazole and analyzed by SDS–polyacrylamide gel electrophoresis.

### ATPase activity

Assays were performed at 25°C in 40 mM Hepes (pH 7.6), 50 mM KCl, and 11 mM Mg acetate, with 5 μM DnaK and an ATP regenerator system: 1 mM ATP, 2 mM phosphoenolpyruvate, 0.25 mM NADH^+^, pyruvate kinase (15 μg/ml), and lactate dehydrogenase (17 μg/ml). Reactions were monitored measuring the absorbance decay at 340 nm in a Cary spectrophotometer (Varian). DnaJ and ubiquitin were used in a concentration of 0.5 and 5 μM, respectively.

### MD simulations

MD simulations were performed using the NAMD 2.10 software (*47*) with the CHARMM36 protein force field in explicit water (described with the TIP3P-CHARMM parameters) (*48*). All systems were minimized and equilibrated before production runs were launched in the constant pressure–constant temperature ensemble. Two different systems were considered for our approach. First, the eukaryotic homolog of DnaJ, HSP40 YnaJ, was crystallized in complex with its cognate substrate GWLYEIS (PDB: 1NLT). We identified with a sequence alignment the amino acid segment of the stretched ubiquitin recognized by DnaJ in our experiments. We constructed by homology the equivalent complex formed by Hsp40 and the ubiquitin segment GKQLEDG, found at positions 47 to 53 in the ubiquitin sequence (PDB: 1UBQ). The structure of the complex was refined with Whatif (http://swift.cmbi.ru.nl/) and solvated in a large cubic box (12-nm side). A production run of 20 ns was used to define the most probable backbone dihedral angles of the client protein fragment. Second, in another set of simulations, the same fragment, capped by two additional ubiquitin residues on the N- and C-terminal side (FAGKQLEDGRT), was generated in an extended conformation, whose end-to-end direction was initially aligned to the vertical axis of a 3 nm × 3 nm × 6 nm box. The C_α_ atom of the first residue was fixed, whereas a constant force (ranging between 50 and 300 pN) was applied to the C_α_ atom of the last residue in the vertical direction. After a long equilibration, at each force, five runs were propagated for 30 ns to estimate the average distribution of backbone dihedral angles for each residue of the consensus binding sequence. The probabilities of observing, for each residue, the most probable backbone dihedral angles in the absence of force were summed to obtain the free-energy cost to bring the ubiquitin backbone dihedral angles from their distributions at this force to their values in the DnaJ-ubiquitin complex.

### Binding rate calculation

We implemented a kinetic model based on ordinary differential equations to evaluate the association and dissociation constants of the chaperone to the polyprotein placed under force, using Python 2.7 and the SciPy library. Within the framework of this model, we considered five different states for the substrate $({\mathrm{Ubiquitin}}^{\mathrm{folded}}$, $U_{free}^{extended}$, $U_{bound}^{extended}$, $U_{free}^{collapsed}$, and $U_{bound}^{collapsed})$ that can exchange over time, depending on the force regime (force-pulse or quench). Only two rate constants were maintained constant to their reference value in the absence of chaperone: $(U^{\mathrm{folded}}$→$U_{free}^{extended}$ and $\begin{array}{c} collapsed\\ free \end{array}$→$U^{\mathrm{folded}})$, whereas all other binding constants were optimized to provide the best overall fit to the experimental data. The goodness of the fit in each case was estimated from χ^2^ values. Further experimental details are provided in figs. S11 and S12.

## Supplementary Material

http://advances.sciencemag.org/cgi/content/full/4/2/eaaq0243/DC1

## SUPPLEMENTARY MATERIALS

Supplementary material for this article is available at http://advances.sciencemag.org/cgi/content/full/4/2/eaaq0243/DC1 Supplementary Information Methods fig. S1. The collapse dynamics of the extended polyubiquitin chain is slowed down upon DnaJ binding. fig. S2. Specifically designed mutations in ubiquitin impair protein folding. fig. S3. DnaJ blocks I27 refolding by binding to the collapsed (and not the native or extended) states. fig. S4. Energetic cost of changing the dihedral angles upon DnaJ binding to the stretched ubiquitin. fig. S5. Ramachandran plots for each ubiquitin fragment residue that interacts with DnaJ as a function of the pulling force. fig. S6. The refolding kinetics of ubiquitin in the presence of DnaK cannot be captured by a single exponential. fig. S7. The ATPase activity of DnaK is not increased upon incubation with ubiquitin. fig. S8. DnaK recognizes the collapsed states of I27, blocking refolding. fig. S9. Calculation of the binding (*k*_on_) and unbinding (*k*_off_) rate constants reveals that DnaJ and DnaK associate to different conformations of ubiquitin and I27. fig. S10. Calculation of the binding $\left(k_{\text{on}}^{\text{extended}}\right)$ and unbinding $\left(k_{\text{off}}^{\text{extended}}\right)$ constants of DnaJ to the extended conformations of ubiquitin. fig. S11. BSA does not affect the kinetics of ubiquitin refolding. fig. S12. Independent addition of the different components of the KJE system improves the DnaK-mediated refolding of ubiquitin. fig. S13. The complete DnaKJE system significantly enhances the rate and the extent of I27 refolding. fig. S14. The DnaK system refolds the folding-inefficient titin (Z1)_8_ polyprotein. fig. S15. Proposed sequences can be recognized by DnaK. table S1. Summary of the kinetic parameters obtained after fitting for ubiquitin and I27. Reference (*49*)
