# Supplementary material for: The force-dependent mechanism of DnaK-mediated mechanical folding
Source: Sci Adv. 2018 Feb 9;4(2):eaaq0243. doi: 10.1126/sciadv.aaq0243 (PMC5817926; doi:10.1126/sciadv.aaq0243)
Supplement: http://advances.sciencemag.org/cgi/content/full/4/2/eaaq0243/DC1 [file supp_4_2_eaaq0243__index.html]

Science Advances | Science Advances

## Supplementary Materials

**This PDF file includes:**

- Supplementary Information Methods
- fig. S1. The collapse dynamics of the extended polyubiquitin chain is slowed down upon DnaJ binding.
- fig. S2. Specifically designed mutations in ubiquitin impair protein folding.
- fig. S3. DnaJ blocks I27 refolding by binding to the collapsed (and not the native or extended) states.
- fig. S4. Energetic cost of changing the dihedral angles upon DnaJ binding to the stretched ubiquitin.
- fig. S5. Ramachandran plots for each ubiquitin fragment residue that interacts with DnaJ as a function of the pulling force.
- fig. S6. The refolding kinetics of ubiquitin in the presence of DnaK cannot be captured by a single exponential.
- fig. S7. The ATPase activity of DnaK is not increased upon incubation with ubiquitin.
- fig. S8. DnaK recognizes the collapsed states of I27, blocking refolding.
- fig. S9. Calculation of the binding (*k*on) and unbinding (*k*off) rate constants reveals that DnaJ and DnaK associate to different conformations of ubiquitin and I27.
- fig. S10. Calculation of the binding (kextendedon) and unbinding (kextendedoff ) constants of DnaJ to the extended conformations of ubiquitin.
- fig. S11. BSA does not affect the kinetics of ubiquitin refolding.
- fig. S12. Independent addition of the different components of the KJE system improves the DnaK-mediated refolding of ubiquitin.
- fig. S13. The complete DnaKJE system significantly enhances the rate and the extent of I27 refolding.
- fig. S14. The DnaK system refolds the folding-inefficient titin (Z1)8 polyprotein.
- fig. S15. Proposed sequences can be recognized by DnaK.
- table S1. Summary of the kinetic parameters obtained after fitting for ubiquitin and I27.
- Reference (*49*)

Download PDF

**Files in this Data Supplement:**

- Adobe PDF - aaq0243\_SM.pdf
