# Supplementary material for: The force-dependent mechanism of DnaK-mediated mechanical folding
Source: Sci Adv. 2018 Feb 9;4(2):eaaq0243. doi: 10.1126/sciadv.aaq0243 (PMC5817926; doi:10.1126/sciadv.aaq0243)
Supplement: http://advances.sciencemag.org/cgi/content/full/4/2/eaaq0243/DC1 [file aaq0243_SM.pdf]

Science Advances

AAAS

advances.sciencemag.org/cgi/content/full/4/2/eaaq0243/DC1

Supplementary Materials for

The force-dependent mechanism of DnaK-mediated mechanical folding

Judit Perales-Calvo, David Giganti, Guillaume Stirnemann, Sergi Garcia-Manyes

Published 9 February 2018, *Sci. Adv.* **4**, eaaq0243 (2018)
DOI: 10.1126/sciadv.aaq0243

This PDF file includes:

- Supplementary Information Methods
- fig. S1. The collapse dynamics of the extended polyubiquitin chain is slowed down upon DnaJ binding.
- fig. S2. Specifically designed mutations in ubiquitin impair protein folding.
- fig. S3. DnaJ blocks I27 refolding by binding to the collapsed (and not the native or extended) states.
- fig. S4. Energetic cost of changing the dihedral angles upon DnaJ binding to the stretched ubiquitin.
- fig. S5. Ramachandran plots for each ubiquitin fragment residue that interacts with DnaJ as a function of the pulling force.
- fig. S6. The refolding kinetics of ubiquitin in the presence of DnaK cannot be captured by a single exponential.
- fig. S7. The ATPase activity of DnaK is not increased upon incubation with ubiquitin.
- fig. S8. DnaK recognizes the collapsed states of I27, blocking refolding.
- fig. S9. Calculation of the binding (k_{on}) and unbinding (k_{off}) rate constants reveals that DnaJ and DnaK associate to different conformations of ubiquitin and I27.
- fig. S10. Calculation of the binding ($k_{on}^{extended}$) and unbinding ($k_{off}^{extended}$) constants of DnaJ to the extended conformations of ubiquitin.
- fig. S11. BSA does not affect the kinetics of ubiquitin refolding.
- fig. S12. Independent addition of the different components of the KJE system improves the DnaK-mediated refolding of ubiquitin.
- fig. S13. The complete DnaKJE system significantly enhances the rate and the extent of I27 refolding.
- fig. S14. The DnaK system refolds the folding-inefficient titin $(Z1)_8$ polyprotein.
- fig. S15. Proposed sequences can be recognized by DnaK.

- table S1. Summary of the kinetic parameters obtained after fitting for ubiquitin and I27.
- Reference (*49*)

Supplementary Information Methods

Molecular Dynamics Simulations

- Generalities

MD simulations were performed using the NAMD 2.10 software(*47*) with the CHARMM36 protein forcefield in explicit water(*48*) (described with the TIP3P-CHARMM parameters). All systems were neutralized with Na^+ and Cl^- ions. Two different systems were considered for our approach. First, the eukaryotic homolog of DnaJ, HSP40 YnaJ which has been crystallized in complex with his cognate substrate GWLYEIS (PDB: 1NLT). We identified with a sequence alignment the amino acid segment of the stretched ubiquitin recognized by DnaJ in our experiments. We constructed by homology the equivalent complex formed by HSP40 and the ubiquitin segment GKQLEDG, found at the position 47-53 in the ubiquitin sequence (PDB:1UBQ). The structure of the complex was refined with Whatif (http://swift.cmbi.ru.nl/) and solvated in a large cubic box (12-nm side, system A). Second, in another set of simulations, the same fragment, capped by two additional ubiquitin residues on the N-terminal and C-terminal side (FAGKQLEDGRT) was generated in an extended conformation, whose end-to-end direction was initially aligned to the vertical axis of a 3*3*6 nm^3 box (system B). The C_α atom of first residue was fixed, while a constant force (ranging between 50 and 300 pN) was applied to the C_α atom of the last residue in the vertical direction.

- Simulation procedure

The atomic positions were first minimized for 2,000 steps using the steepest-descent algorithm of NAMD. All subsequent simulations were run in the NPT ensemble (300 K, 1 bar), using the Langevin thermostat (0.1/ps) and the Langevin barostat of NAMD. Long-range electrostatics were treated using particle-mesh Ewald with a grid size of 1.0 $Å^{-1}$. Simulation timestep was 2 fs and bonds between hydrogen and heavy atoms were maintained rigid.

System A: after minimization, the ubiquitin fragment was frozen and DnaJ was relaxed for 2 ns at ambient temperature and pressure. Subsequently, the constraint on the ubiquitin fragment was removed and the system was equilibrated for 2 ns. A production run of 20 ns was performed to determine the most probable backbone dihedral angles of the ubiquitin fragment in the complex.

System B: after minimization, the system was equilibrated under ambient conditions and at the respective forces for 40 ns. Subsequently, 5 runs of 30-ns each were performed at each force.

Circular Dichroism experiments

Far UV spectra (200-260 nm) of the monomeric form of WT ubiquitin and the two soluble mutants were recorded on a Jasco J-810 spectropolarimeter at 22 ºC in 10 mM KH_2PO_4. The concentration of the protein was 10 μM and 20 individual spectra were accumulated to obtain the average read-out in each case.

Supplementary Figures

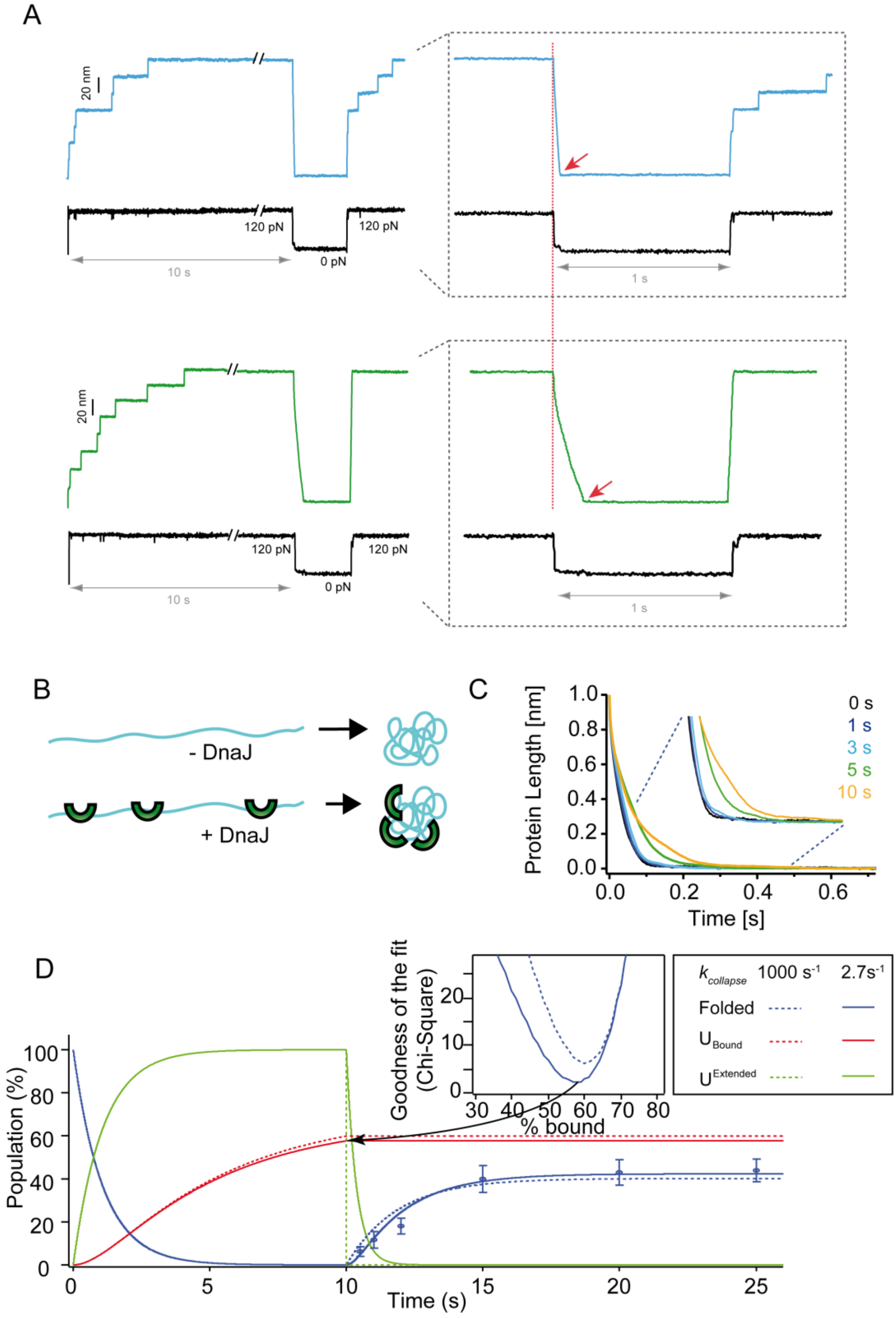

fig. S1. The collapse dynamics of the extended polyubiquitin chain is slowed down upon DnaJ binding. (**A**) Experimental trajectories showing that a long exposure time to DnaJ (10 s) triggers binding to the extended ubiquitin (green trace) and a subsequent slower collapse of the chain (red arrow) as compared to the situation in the absence of DnaJ (blue). (**B**) Schematic representation of the binding mechanism. (**C**) The average ubiquitin collapse

trajectory (down to 0 force) is significantly slowed down upon increasing the time, t_{ext}, the protein is unfolded and exposed to 5μM DnaJ for t_{ext} varying 1-10s. (**D**) Refolding of free ubiquitin domains after an exposure time of 10 s at 120 pN (blue circles) is slowed down because of the slower collapse in the presence of DnaJ. This is confirmed by the analysis of the experimental data using a kinetic model and a multiparametric fit described in fig. S9. Within the framework of this model, a fit of the data (dashed blue line) using a fixed folding rate identical to that measured in the absence of DnaJ, and a very fast and fixed $k_{collapse}$=1000 s^{-1}, cannot capture the experimental data because it significantly deviates at short quenching times (χ^2= 6.00, inset). Instead, introducing an additional parameter $k_{collapse}$ during the fitting procedure reproduces the experimental values better (χ^2= 2.26, blue line). This fit leads to a value of $k_{collapse}$ = 2.73 s^{-1}, which is in very good agreement with the collapse timescale measured in (**C**). Noticeably, the obtained fraction of bound domains after an exposure time of 10 s remains unchanged (~60% at this force), therefore suggesting that the interpretation of the experimental data is not affected by the interplay between slow collapse and refolding at short quench times.

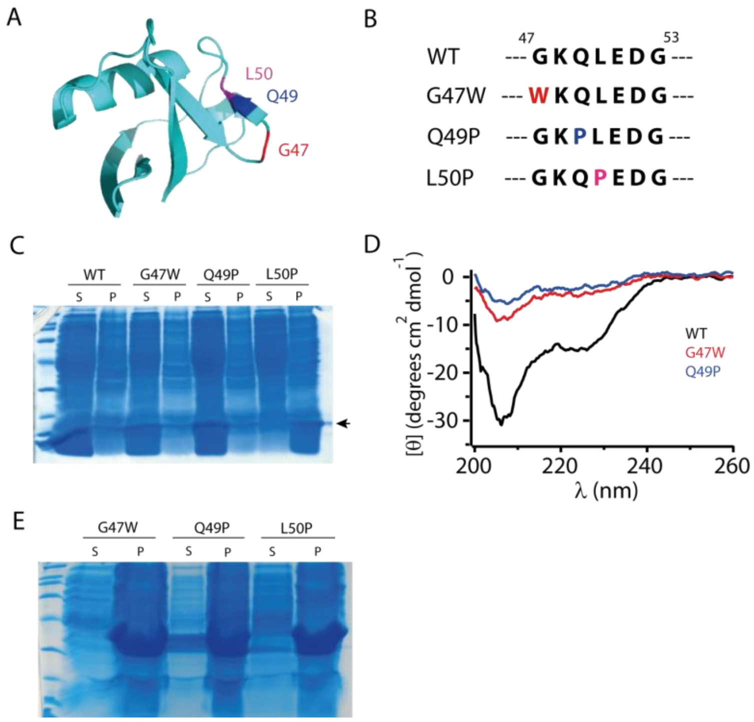

fig. S2. Specifically designed mutations in ubiquitin impair protein folding. (A) Mutated residues in the Ubiquitin structure (PDB: 1UBQ) that have been selected for mutation in an attempt to disrupt the consensus sequence for DnaJ binding (according to Kota *et al.*, *PNAS*, 2009)(*13*). **(B)** Specific single point Ubiquitin mutants. (**C**) Solubility test for Ubi WT and its mutants. After cell lysis, the WT protein and the mutants G47W and Q49P are soluble. However, the protein corresponding to the mutant L50P aggregates and appears in the pellet (arrow). (S: supernatant; P: pellet). **(D)** Circular dichroism spectra for the WT protein (black) and the two soluble monomeric mutants (G47W, in red, and Q49P, in blue), demonstrating the loss in protein structure triggered by the inserted mutations. **(E)** Solubility test for the tetramers of the three mutants: $(G47W)_4$, $(Q49P)_4$ and $(L50P)_4$, evidencing that the polyproteins of the three mutants aggregate (being present only in the pellet).

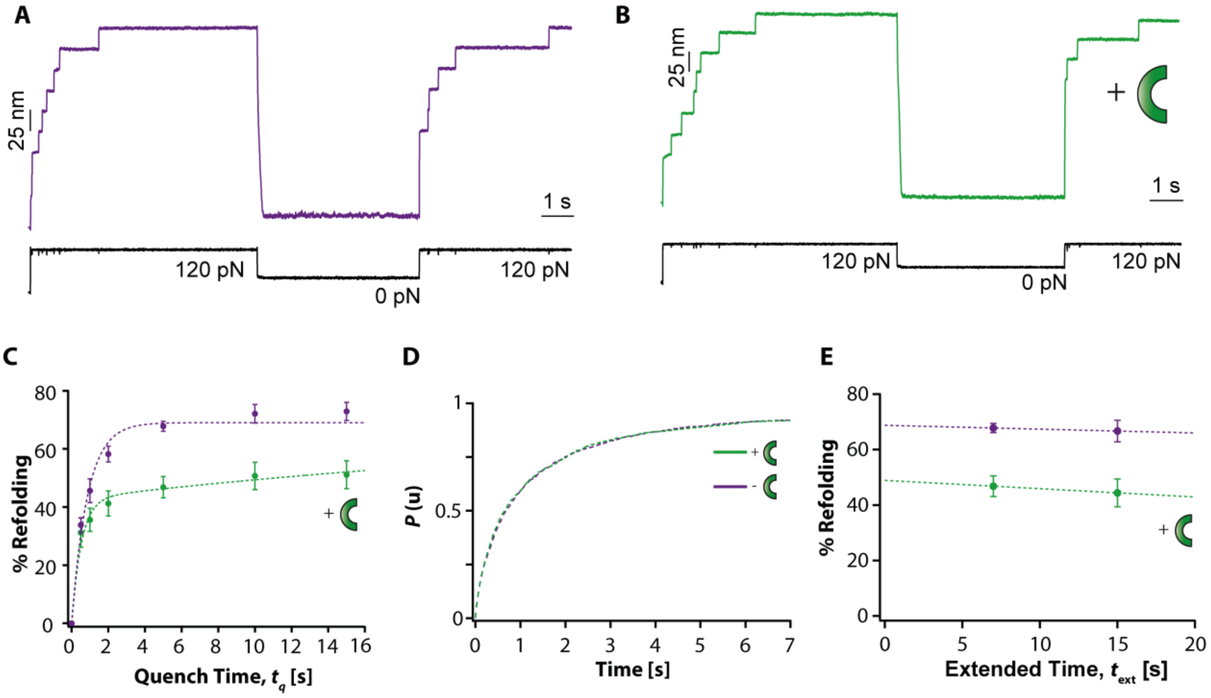

fig. S3. DnaJ blocks I27 refolding by binding to the collapsed (and not the native or extended) states. (**A**) The refolding of an $(I27)_8$ polyprotein is largely quantitative after a long t_q = 5 seconds, fingerprinted by a large population of 25 nm steps in the *test* pulse. (**B**) The refolding yield is significantly decreased upon addition of 5 μM DnaJ. (**C**) The I27 refolding kinetics, obtained from varying t_q within a range spanning 0.5-15 s, shows that the ratio of refolding domains is substantially lower in the presence of 5 μM DnaJ. In the absence of DnaJ, t_q = 0.5s, *n*=33 individual trajectories; t_q = 1s, *n*=28; t_q = 2s, *n*=45; t_q = 5s, *n*=54; t_q = 10s, *n*=23; t_q = 15s, *n*=24. In the presence of DnaJ, t_q = 0.5s, *n*=23; t_q = 1s, *n*=29; t_q = 2s, *n*=31; t_q = 5s, *n*=39; ; t_q = 10s, *n*=34; t_q = 15s, *n*=22. Dashed lines correspond to mutiparametric fits based on the kinetic model presented in fig. S9. (**D**) The unfolding kinetics of I27 is not varied in the presence (k_u = 1.03 ± 0.06 s^{-1}, *n*=146 unfolding trajectories) or in the absence (k_u = 1.06 ± 0.04 s^{-1}, *n*=231 unfolding trajectories) of 5 μM DnaJ, suggesting that DnaJ does not bind to the native state of I27. (**E**) Similarly, varying t_{ext} in the presence of DnaJ (t_{ext} = 7s, *n*=39; t_{ext} = 15s, *n*=24) does not have a significant effect on I27 refolding, concluding that DnaJ, in contrast to the case of ubiquitin, does not bind to the unfolded and extended state of I27 (lines are to guide the eye). These experiments thus point towards a scenario where DnaJ binds to the collapsed states of I27 instead.

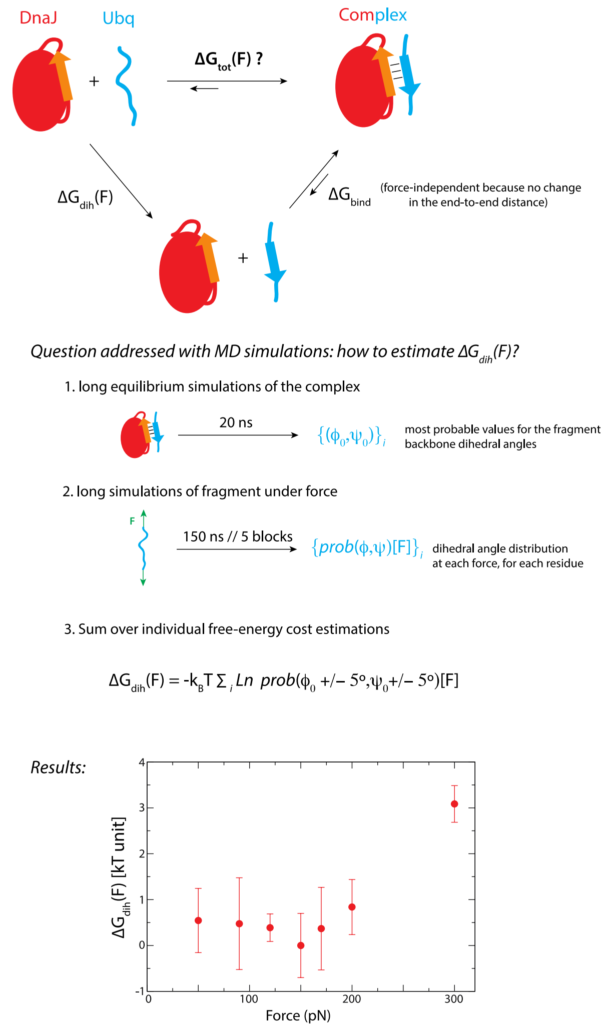

fig. S4. Energetic cost of changing the dihedral angles upon DnaJ binding to the stretched ubiquitin. In our approach, we assume that the free-energy of interaction between the ubiquitin fragment and DnaJ, $\Delta G_{tot}(F)$, results at each force from the sum of the cost to bring each backbone dihedral angle at this force to its value in the protein complex $\Delta G_{dih}(F)$, and the cost of bringing this pre-arranged conformation in contact with DnaJ ΔG_{bind} (which is assumed to be force-independent as it does not lead to any change in the end-to-end distance, and which is not calculated). At each force, $\Delta G_{dih}(F)$ is calculated as follows: (1) the most probable values for the 5 couples of backbone dihedral angles (ϕ_0, ψ_0) of the ubiquitin consensus sequence (that be defined for LYS 48, GLN 49, LEU 50, GLU 51 and ASP 52) are estimated from a 20-ns simulation of system A (containing DnaJ in complex with the ubiquitin fragment). (2) The probability distributions of (ϕ, ψ) dihedral angles for each of these residues (i.e., their Ramachandran plot) are determined at each force from a set of 5 simulations of 30 ns each, using a discrete grid with a resolution of 3.6º for each angle. The free-energy cost (3) is defined as the sum of the individual free-energy costs for each residue, defined from the probability of observing (ϕ_0 +/- 5º, ψ_0+/- 5º). Error bars correspond to the standard deviations observed among the 5 independent runs at each force, and the data is normalized with respect to the lowest value (observed at 150 pN).

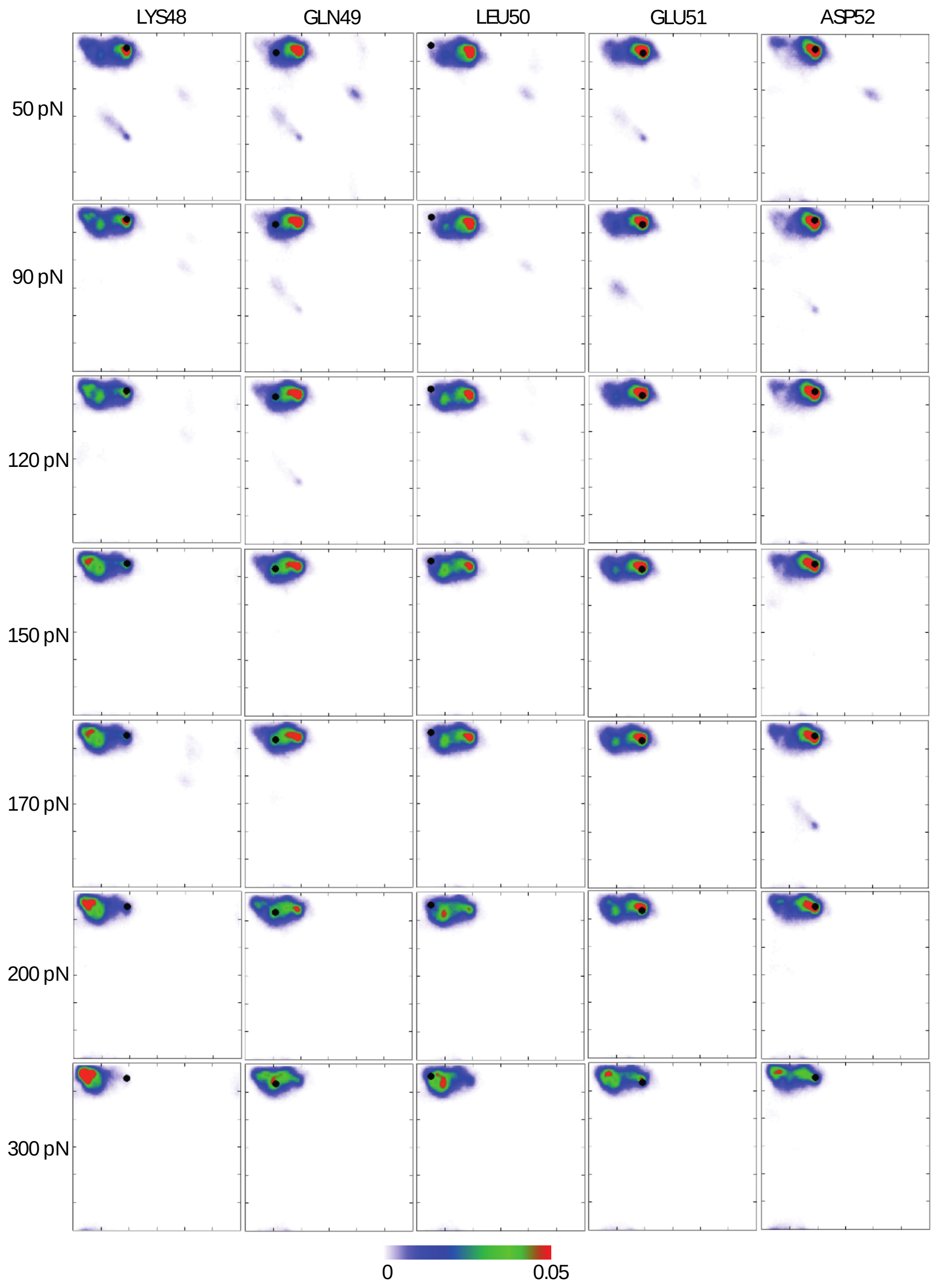

fig. S5. Ramachandran plots for each ubiquitin fragment residue that interacts with DnaJ as a function of the pulling force. (Colour code, from least probable in blue to most probable in red). Black dots indicate the most probable value in the DnaJ-bound complex, in the absence of force.

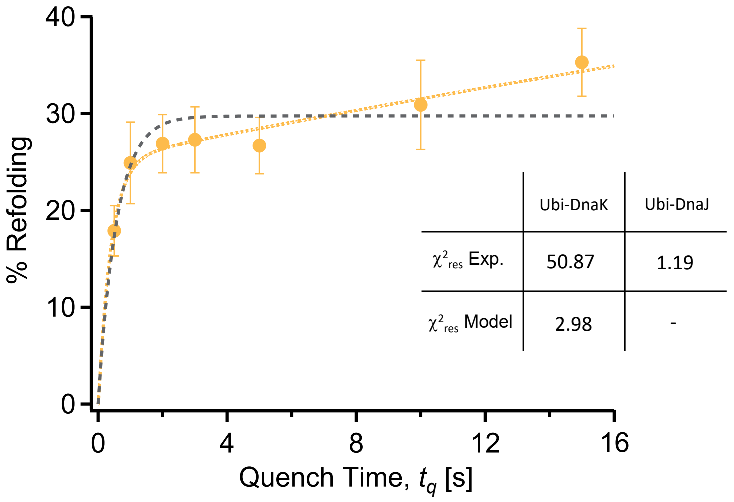

fig. S6. The refolding kinetics of ubiquitin in the presence of DnaK cannot be captured by a single exponential. Fitting a single exponential to the refolding evolution of ubiquitin in the presence of 5 μM DnaK does not capture the experimental data. By contrast, fitting the data with a kinetic model that takes into account the conformation-dependent binding of DnaJ (see fig. S9 and Fig. 4 in the main text) leads to better results, as confirmed by the reduced χ^2 values.

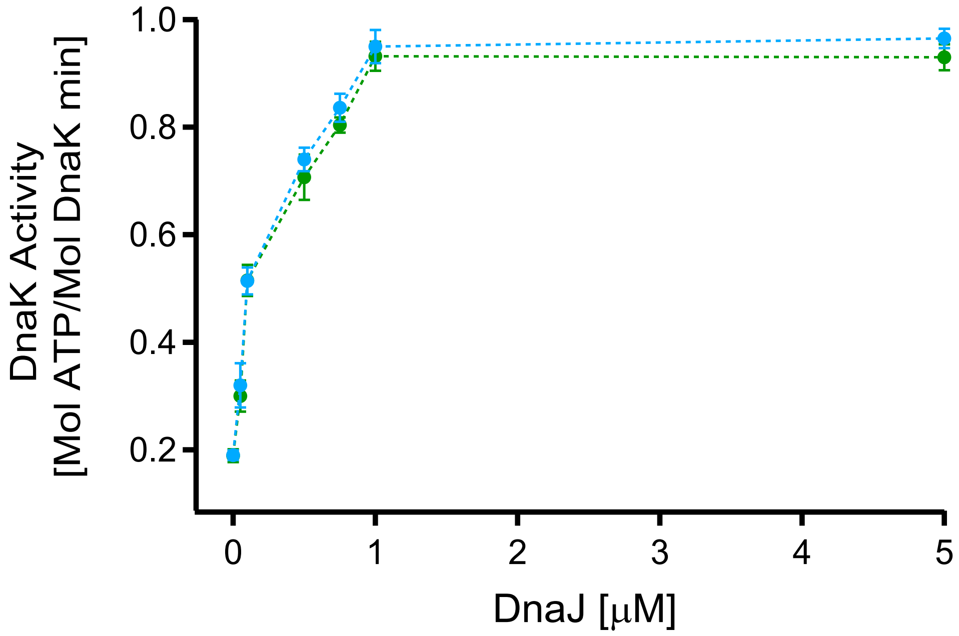

fig. S7. The ATPase activity of DnaK is not increased upon incubation with ubiquitin. The ATPase activity of DnaK was followed during 10-15 min for increasing concentrations of DnaJ (blue dots). The addition of ubiquitin 5 μM (green dots) does not affect DnaK activity, which suggests that the chaperone is not able to recognize and to bind the native conformation of ubiquitin (from *n*= 4 independent experiments).

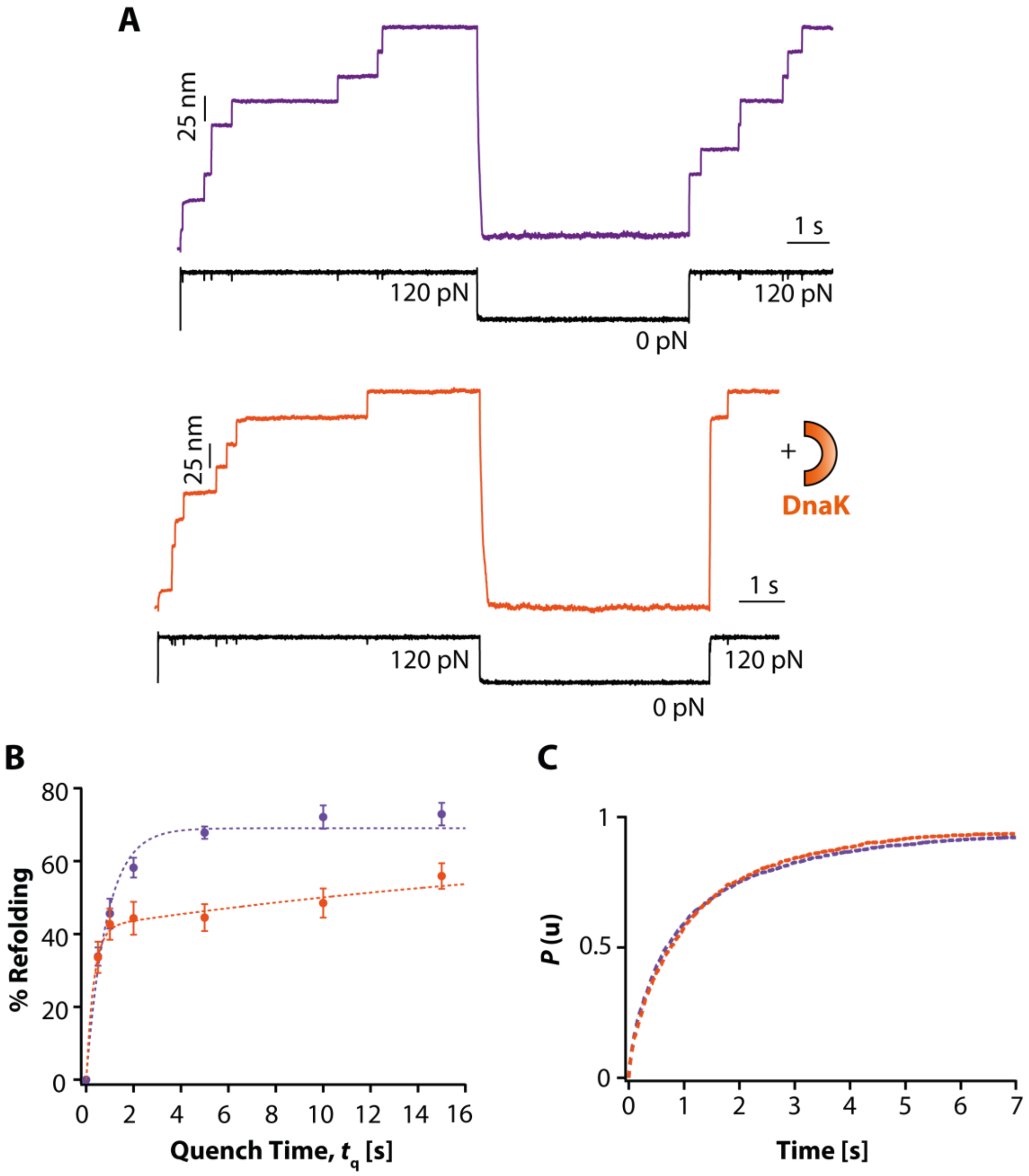

fig. S8. DnaK recognizes the collapsed states of I27, blocking refolding. (**A**) The yield of I27 refolding is significantly impaired in the presence of 5 μM DnaK. (**B**) The reformation kinetics obtained by varying t_q in the range 0.5-15 seconds shows that DnaK partially impairs I27 refolding. Dashed lines correspond to mutiparametric fits based on the kinetic model presented in fig. S9 (t_q = 0.5s, n=27 individual trajectories; t_q = 1s, n=33; t_q = 2s, n=36; t_q = 5s, n=47; t_q = 10s, n=22; t_q = 15s, n=31). (**C**) Like DnaJ, DnaK does not bind to the native state of I27, as confirmed by the unchanged rate of I27 unfolding (k_u = 1.06 ± 0.04 s^{-1}, n=231 unfolding events) in the presence of 5 μM DnaK (k_u = 0.98 ± 0.05 s^{-1} n=155 unfolding trajectories).

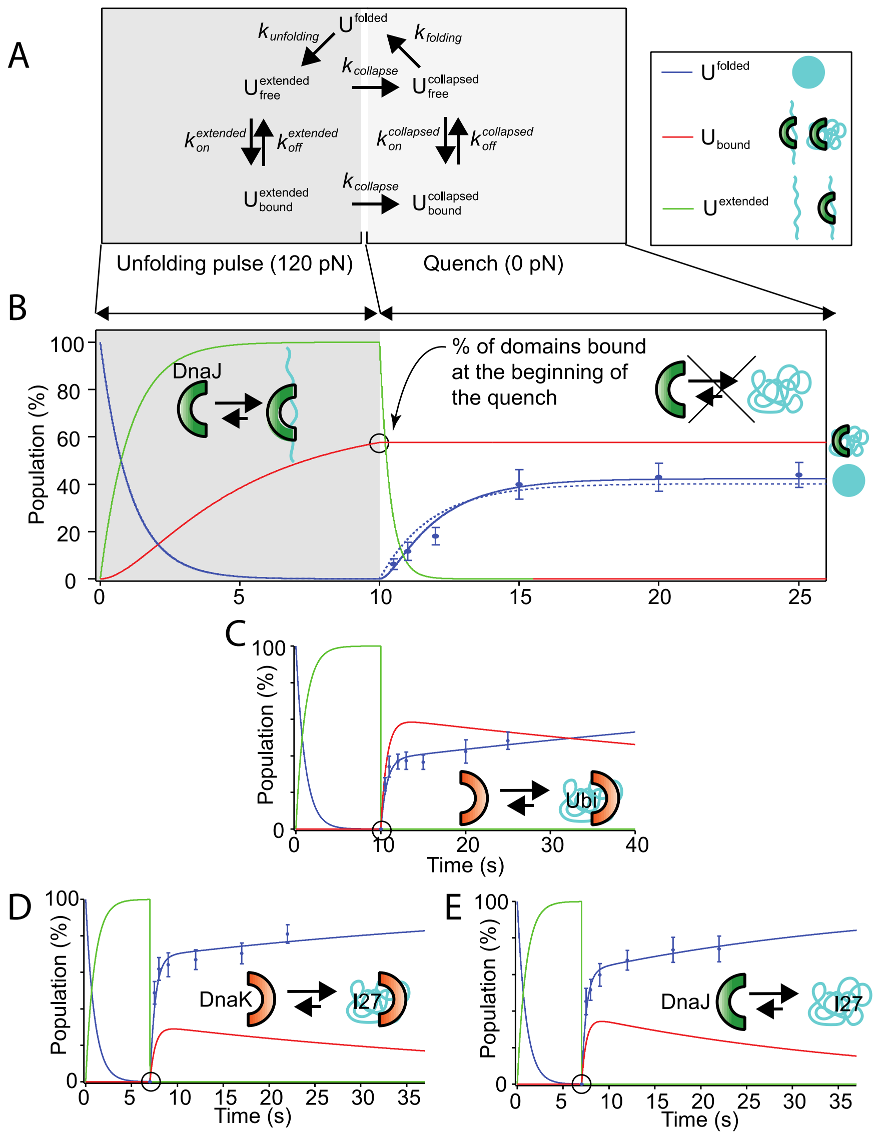

fig. S9. Calculation of the binding (k_{on}) and unbinding (k_{off}) rate constants reveals that DnaJ and DnaK associate to different conformations of ubiquitin and I27. (A) Description of the kinetic model that contains all possible transitions between the five substrate conformations:

$(Ubiquitin^{folded}\ U^{extended}_{free}, U^{extended}_{bound}, U^{collapsed}_{free}, U^{collapsed}_{bound})$. The kinetic model is hence defined by a set of five ordinary differential equations:

$$\frac{d[U^{\text{folded}}]}{dt} = -k_{\text{unfolding}}[U^{\text{folded}}] + k_{\text{folding}}[U^{\text{collapsed}}_{\text{free}}]$$

$$\frac{d[U^{\text{extended}}_{\text{free}}]}{dt} = k_{\text{unfolding}}[U^{\text{folded}}] - k_{\text{collapse}}[U^{\text{extended}}_{\text{free}}] - k^{\text{extended}}_{\text{on}}[U^{\text{extended}}_{\text{free}}][C]_0 + k^{\text{extended}}_{\text{off}}[U^{\text{extended}}_{\text{bound}}]$$

$$\frac{d[U^{\text{collapsed}}_{\text{free}}]}{dt} = k_{\text{collapse}}[U^{\text{extended}}_{\text{free}}] - k_{\text{folding}}[U^{\text{collapsed}}_{\text{free}}] - k^{\text{collapsed}}_{\text{on}}[U^{\text{collapsed}}_{\text{free}}][C]_0 + k^{\text{collapsed}}_{\text{off}}[U^{\text{collapsed}}_{\text{bound}}]$$

$$\frac{d[U^{\text{extended}}_{\text{bound}}]}{dt} = k^{\text{extended}}_{\text{on}}[U^{\text{extended}}_{\text{free}}][C]_0 - k^{\text{extended}}_{\text{off}}[U^{\text{extended}}_{\text{bound}}] - k_{\text{collapse}}[U^{\text{extended}}_{\text{bound}}]$$

$$\frac{d[U^{\text{collapsed}}_{\text{bound}}]}{dt} = k^{\text{collapsed}}_{\text{on}}[U^{\text{collapsed}}_{\text{free}}][C]_0 - k^{\text{collapsed}}_{\text{off}}[U^{\text{collapsed}}_{\text{bound}}] + k_{\text{collapse}}[U^{\text{extended}}_{\text{bound}}]$$

where $[C]_0$ is the concentration of chaperone present in solution. At the beginning of the unfolding pulse, all domains are in the U^{folded} state. At the end of the unfolding pulse, we assumed that all bound (free) domains remain associated (free) with the chaperone as the chain collapsed ($U^{extended}_{bound} = U^{collapsed}_{bound}$ and $U^{extended}_{free} = U^{collapsed}_{free}$). We solved the differential equations describing the model with numerical integration (Python2.7, scipy library) to estimate the evolution of the system over time. In most cases, the rapid transition from the extended state to the collapsed state upon quenching the pulling force occurs almost instantaneously within the experimental timescale (~100 ms), and hence a corresponding high collapse rate was set for such an extended to collapse transition. The only exception is the DnaJ/Ubi system, where the binding of DnaJ to the extended state results in a significantly slower collapse, on a timescale comparable to the refolding kinetics of the collapsed protein (fig. S1). Therefore, only in that specific case, the model included the collapse rate as a floating parameter that was determined during the fitting procedure described below. Folding (k_{folding}) and unfolding ($k_{\text{unfolding}}$) rates were kept constant at their values measured in the absence of chaperones. Finally, AFM experiments on individual polyproteins typically show a maximum refolding yield capped at ~60-80%(*22, 42*). According this experimental observation, we used a normalization factor (1.37 for ubiquitin and 1.45 for I27) so that the refolding percentage for an infinite quench time would be 100% (*42*). Within the framework of our model, any refolding yield lower than 100% would correspond to the binding of the chaperone to the remaining fraction of proteins. **(B) DnaJ + ubiquitin:** During the unfolding pulse at 120 pN (first 10 s), the population of folded domains decreases exponentially over time (blue line between 0 and 10 s, $k_{\text{unfolding}} = 0.93\ s^{-1}$), resulting in extended conformations that expose cryptic sites recognized by DnaJ. We estimated the on- and off- rates by varying the time the protein was extended, t_{ext}, cf. fig. S10. At the end of the unfolding pulse, only a fraction of the extended domains is bound. The chain collapses into a compact, non-folded conformation at a rate k_{collapse}, and only the free (unbound) domains are able to refold at a fixed rate $k_{\text{folding}} = 0.54\ s^{-1}$. The experimental data corresponding to the percentage of refolded domains at different quench times are added as blue symbols. To fit the refolding data, we optimized the goodness of the fit (χ^2) to find the best three parameters: $k^{collapsed}_{on}$, $k^{collapsed}_{off}$ and the initial condition $U^{extended}_{bound}$ (marked with a black circle). In our method, we searched for the two rate

constants for all possible $U^{extended}_{bound}$ varying from 0 to 100% with an increment of 1%. In the corresponding experimental conditions (DnaJ + ubiquitin stretched at 120 pN for 10 s), the optimal fit (dotted blue line) corresponds to a scenario where 60% of the Ubiquitin domains are bound after 10 s (black circle). The fit was significantly better when k_{collapse} was not set at a fixed, very fast value (solid blue line), but was instead left as a free parameter during the fitting procedure, resulting in a value of 2.73 s^{-1} (this fit is also shown in fig. S1). This observation is in line with the experimental evidence that for this specific DnaJ/Ubi system, collapse was slowed down as compared to the other protein/chaperone combinations. (**C**) Using the same approach, we measured the evolution of each force-induced protein conformation of ubiquitin in the presence of DnaK, and similarly of **(D, E)** I27 in the presence of (**D**) DnaK and (**E**) DnaJ. For clarity, we represent in these plots the evolution of the states $I27^{extended} = I27^{extended}_{free} + I27^{extended}_{bound}$ in green, $27_{bound} = I27^{extended}_{bound} + I27^{collapsed}_{bound}$) in red, and folded domains in blue.
Within the framework of our model, the deviation from single exponential refolding kinetics is quantitatively explained by the competition between folding and association and/or dissociation during the quench period. The best fits in c, d and e were obtained for $I27^{extended}_{bound}$ < 0.1% and $Ubi^{extended}_{bound}$ < 0.1% (black circle), indicating that binding only occurs in the collapsed state (and not in the extended state). These three chaperone/substrate combinations display a behavior that is radically different from that involving binding of DnaJ to the extended conformation of ubiquitin (**B**).

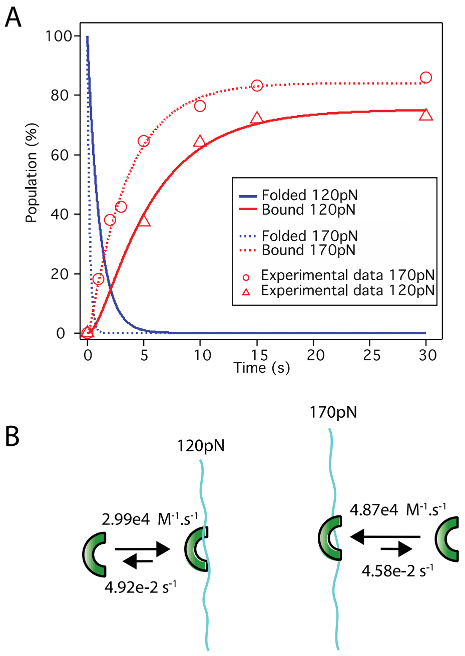

fig. S10. Calculation of the binding $(k_{\mathrm{on}}^{\mathrm{extended}})$ **and unbinding** $(k_{\mathrm{off}}^{\mathrm{extended}})$ **constants of DnaJ to the extended conformations of ubiquitin. (A)** Evolution of the refolding yield of ubiquitin following a quench time of 5 s, and after having been held in the extended state at different forces (120 pN and 170 pN), and for different exposure times (t_{ext} = 0-30 s). The experimental data is fitted within the framework of the kinetic model presented in fig. S9, with $k_{on}^{extended}$, $k_{off}^{extended}$ as free parameters. (**B**) The resulting binding constants ($k_{on}^{extended}$) estimated at 120 pN and 170 pN suggest that the association rate is force-dependent, while the off-rate ($k_{off}^{extended}$) shows little dependence with the stretching force.

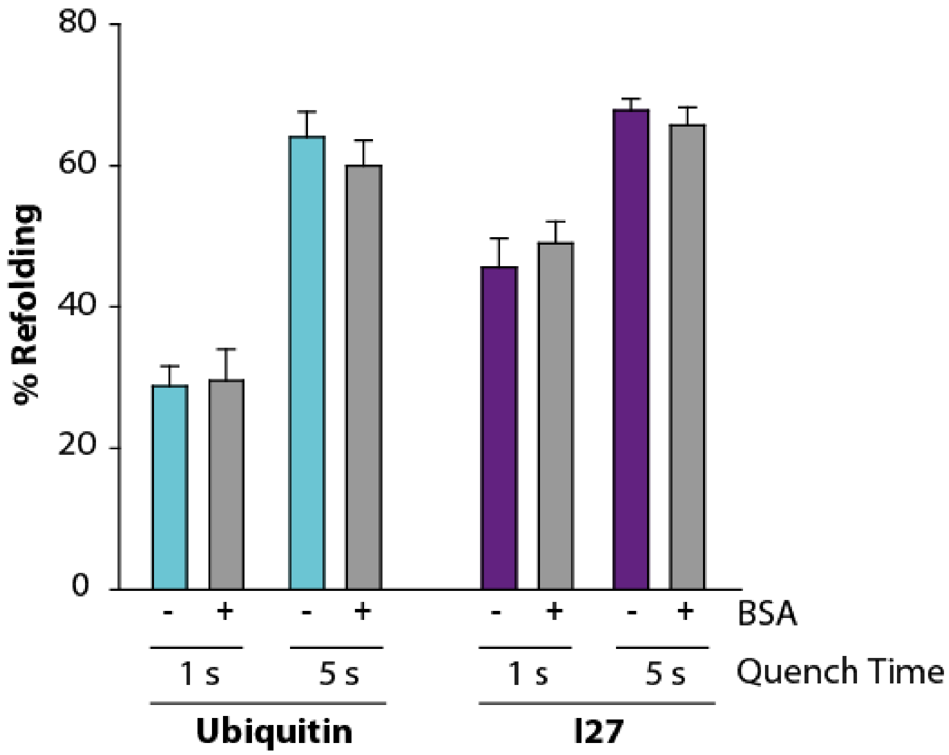

fig. S11. BSA does not affect the kinetics of ubiquitin refolding. The presence of 15 μM BSA does not significantly affect (p >0.5) the refolding kinetics of neither ubiquitin or I27, either for short (t_q = 1s) nor long (t_q = 5s) quench times. In the case of ubiquitin t_q = 1s, n=56 (no BSA) and n=30 (BSA); for t_q = 5s, n=46 (no BSA) and n=27 (BSA). In the case of I27 t_q = 1s, n=28 (no BSA) and n=27 (BSA); for t_q = 5s, n=54 (no BSA) and n=57 (BSA).

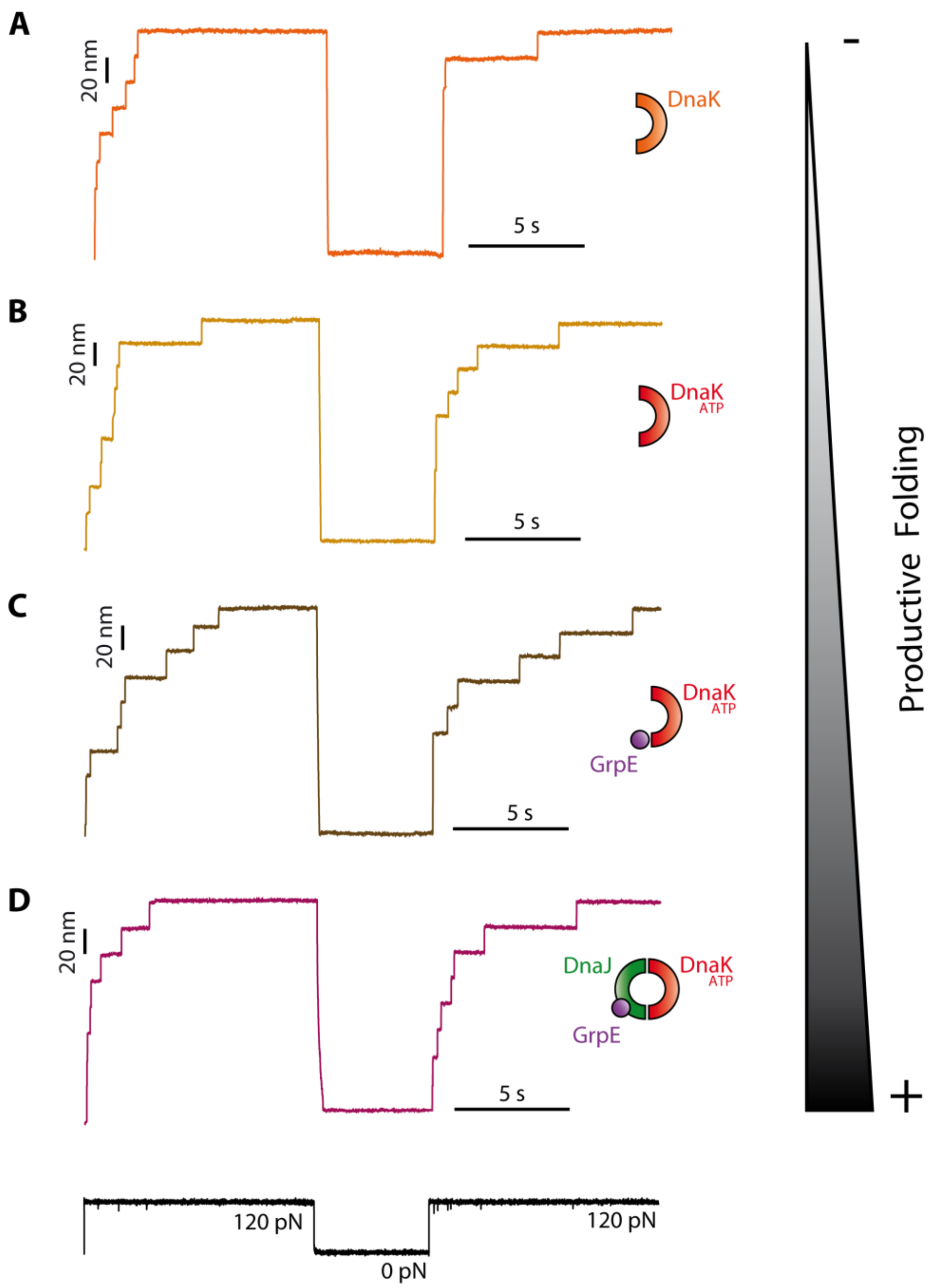

fig. S12. Independent addition of the different components of the KJE system improves the DnaK-mediated refolding of ubiquitin. Individual $(Ubiquitin)_9$ folding trajectories in the presence of DnaK(ADP) (**A**, orange); DnaK(ATP) (**B**, light brown); DnaK(ATP)+GrpE (**C**, dark brown); and DnaK(ATP)+GrpE+DnaJ (**D**, violet). The addition of the different components of the KJE system increases DnaK activity, resulting in an increment of productive refolding (hallmarked by the presence of 20 nm steps in the *test* pulse.

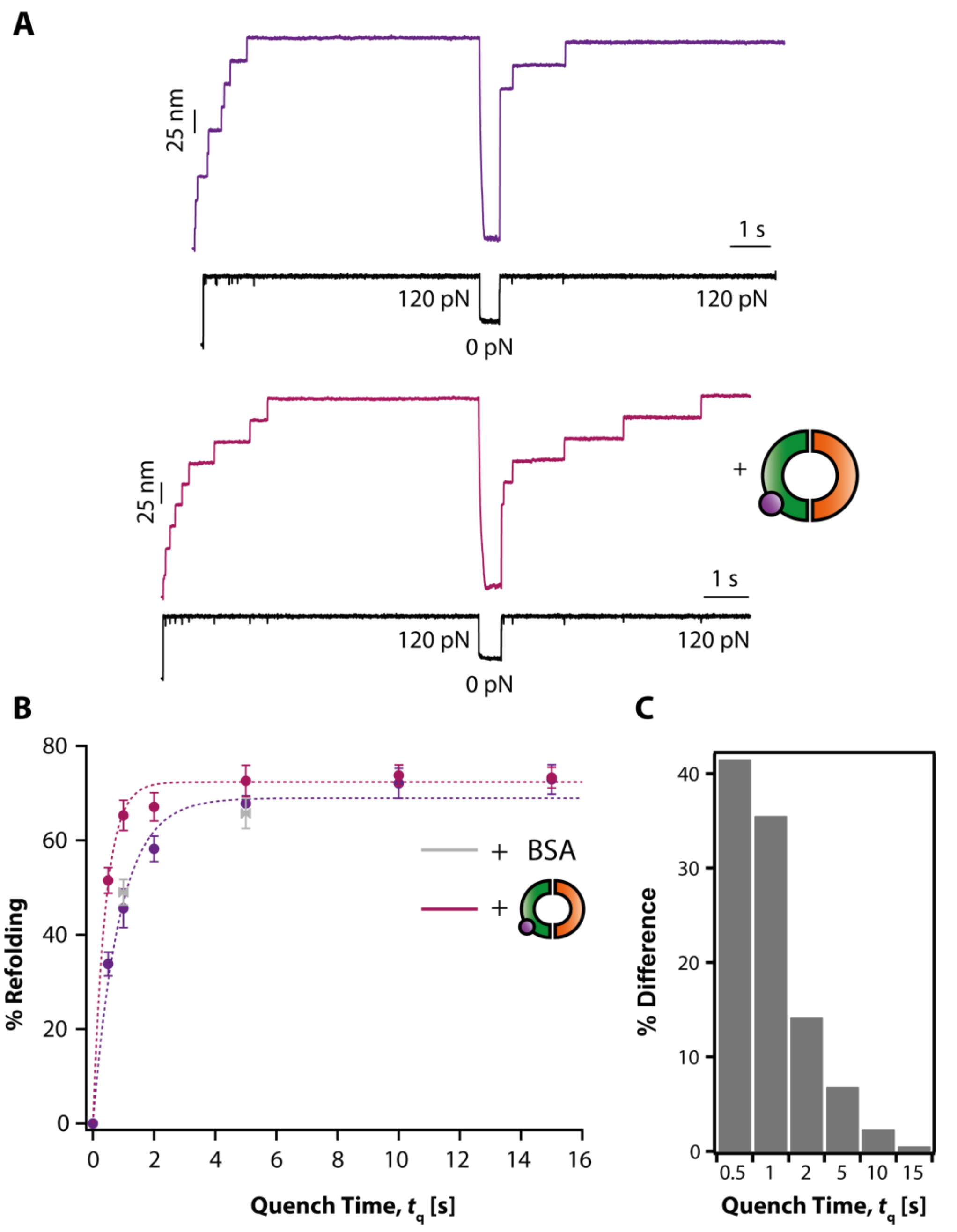

fig. S13. The complete DnaKJE system significantly enhances the rate and the extent of I27 refolding. (**A**) Similar to the case of ubiquitin (Fig. 5 in the main text), addition of the complete DnaK system enhances I27 refolding. (**B**) The refolding kinetics, especially for short t_q values (0.5-2 seconds) is significantly faster ($p < 0.01$) in the presence of the entire DnaK system (t_q = 0.5s, n=52; t_q = 1s, n=35; t_q = 2s, n=28; t_q = 5s, n=29; t_q = 10s, n=32; t_q = 15s, n=38), which not only increases the refolding yield but also accelerates ($k_f = 1.14 \pm 0.1$ s^{-1} *vs* $k_f = 2.42 \pm 0.25$ s^{-1}) the refolding kinetics. **(C)** At short quench times t_q = 0.5-2 s, the folding efficiency is significantly increased [$p < 0.0001$ (t_q = 0.5s); $p < 0.003$ (t_q = 1s); $p = 0.01$ (t_q = 2s)], whereas for longer t_q values, the difference becomes less pronounced ($p > 0.2$).

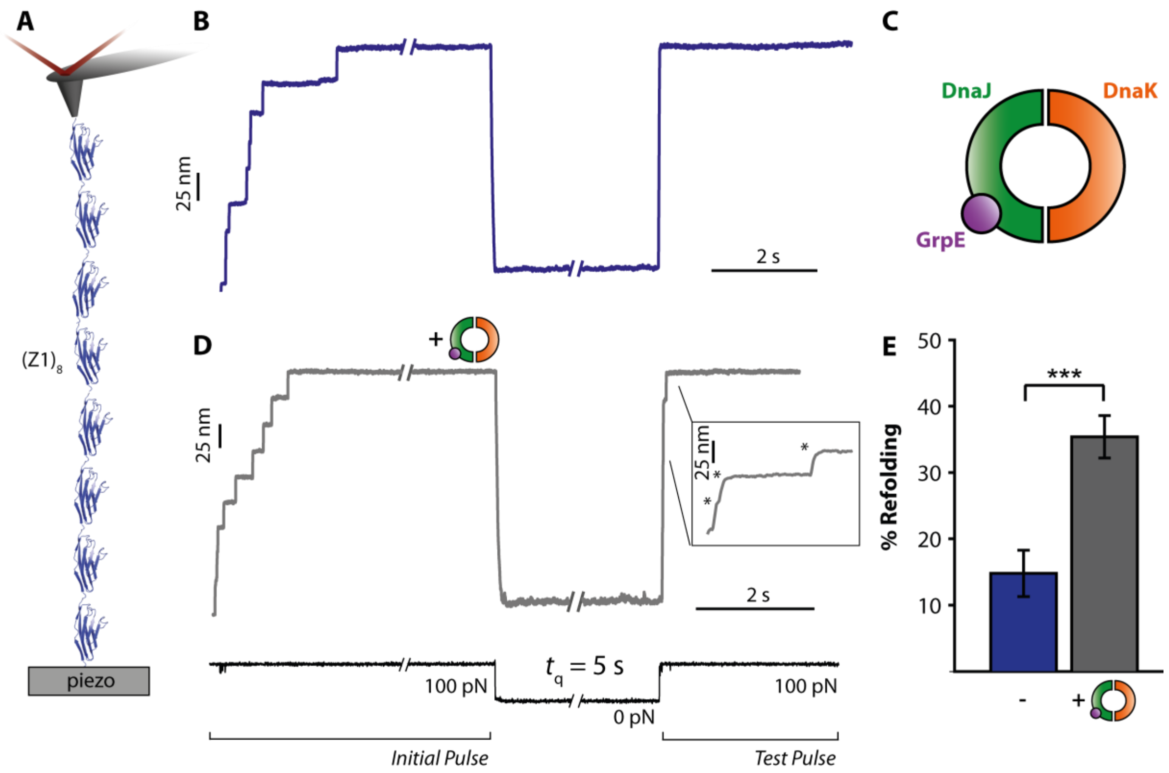

fig. S14. The DnaK system refolds the folding-inefficient titin $(Z1)_8$ polyprotein. (**A**) A polyprotein construct formed of eight identical repeats of the Z1 Ig domain of titin is tethered between a gold substrate and an AFM cantilever tip, which applies a constant stretching force throughout the experiment. (**B**) Using the force-quench approach, upon the application of 100 pN of force, the protein unfolds in a 25 nm steps (*initial* pulse). After 10s, the force is withdrawn for t_q = 5s to trigger protein folding. The folding status of the protein is then probed in the *test* pulse, where the protein is stretched back at a high force of 100 pN. The absence of 25 nm steps in the test pulse fingerprints the absence of refolding of the $(Z1)_8$ polyprotein in this timescale. (**C**) The whole bacterial Hsp70 system is composed of the Hsp70 (DnaK) chaperone (orange), working in synergy with the Hsp40 co-chaperone (DnaJ, green) and the nucleotide exchange factor (GrpE, purple) in the presence of ATP. (**D**) The refolding trajectories of the $(Z1)_8$ polyprotein in the presence of the entire DnaK system (5 μM DnaK + 5 μM DnaJ + 1 μm GrpE+ 5 mM ATP) results in a test pulse featuring 25 nm steps, which fingerprints the successful refolding of Z1. (**E**) Comparison of the folding efficiency of the $(Z1)_8$ polyprotein in the absence (15%, n = 24 trajectories) and the presence of the DnaK system is largely significant (35%, n = 26, $p < 0.0005$), demonstrating that DnaK is able to successfully refold the otherwise folding-inefficient $(Z1)_8$ polyprotein.

I27 (Titin)

UniProtKB: Q8WZ42

LIEVEKPLYG VEVFVGETAH FEIELSEPDV HGQWKLKGQP LAASPDCEII EDGKKHILIL HNCQLGMTGE VSFQAANTKS AANLKVKEL

Ubiquitin

UniProtKB: P0CG48

MQIFVKTLTG KTITLEVEPS DTIENVKAKI QDKEGIPPDQ QRLIFAGKQL EDGRTLSDYN IQKESTLHLV LRLRGG

fig. S15. Proposed sequences can be recognized by DnaK. The residues hallmarked in green for both I27 and Ubiquitin sequences form hydrophobic patches of 4-5 residues that are rich in leucines and isoleucines. These type of patches have been suggested to be recognized by DnaK with high affinity(*11*).

table S1. Summary of the kinetic parameters obtained after fitting for ubiquitin and I27.

		Ubiquitin (120pN)		I27 (150pN)	
		DnaJ*	DnaK	DnaJ	DnaK
binding to the extended state	*% domains bound after 10 s*	58%	<0.1% No apparent k_{on} and k_{off}	<0.1% No apparent k_{on} and k_{off}	<0.1% No apparent k_{on} and k_{off}
binding to the collapsed state	k_{on} (s^{-1})	No apparent k_{on} and k_{off}	1.69×10^{5}	1.42×10^{5}	1.07×10^{5}
	k_{off} (s^{-1})		2.37×10^{-2}	4.72×10^{-2}	2.90×10^{-2}
	K_d *(M)*		1.40×10^{-7}	3.32×10^{-7}	2.71×10^{-7}
Goodness of the fit	χ^2 test	2.27	2.98	1.51	3.29
	p-value	0.95	0.89	0.96	0.77

*data calculated with $k_{collapse} = 2.73\ s^{-1}$ (fig. S9)

Summary of the binding rates calculated from the experimental data and shown in fig. S9 and fig. S10. DnaJ binds to the extended conformation of ubiquitin with a $K_d = k_{off}/k_{on} = 9.4 \times 10^{-7}$ M, which results in the binding of 58% of the domains after an exposure time of 10 s. By contrast, the fitting reveals that DnaK binds exclusively to the collapsed states of I27. The last two rows contain the values associated to goodness of the fit (chi-square, χ^2, and *p*-values).

Noteworthy, all values presented in this table are obtained assuming a constant folding rate for each substrate (I27 and Ubiquitin). Figure S9C,D,E show a rapid increase of the population of refolding domains for short quench times. We reproduced the fitting procedure described in fig. S9 with an additional free parameter, $k_{folding}$. Under these conditions, we observed that the fits were largely improved (lower χ^2 values, all p-values reaching 0.99) and the resulting folding rates are noticeably faster (~1.7 times for DnaK-Ubiquitin, ~1.4 times for DnaJ-I27 and ~1.6 times for DnaK-I27). This effect is hard to quantify for the DnaJ-Ubi system because of the slow collapse induced by DnaJ binding to the extended state of Ubiquitin. It has been proposed that sequence similarity induces enhanced misfolding in homo-polyprotein chains (*49*). In such scenario, it is plausible that chaperone binding to a particular domain protects it from misfolding with adjacent unbound domains by avoiding interdomain non-productive contacts.
